# Engineered Proteins and Chemical Tools to Probe the Cell Surface Proteome

**DOI:** 10.1021/acs.chemrev.4c00554

**Published:** 2025-04-03

**Authors:** Kevin K. Leung, Kaitlin Schaefer, Zhi Lin, Zi Yao, James A. Wells

**Affiliations:** † Department of Pharmaceutical Chemistry, University of California San Francisco, San Francisco, California 94158, United States; ‡ Department of Cellular and Molecular Pharmacology, University of California San Francisco, San Francisco, California 94158, United States

## Abstract

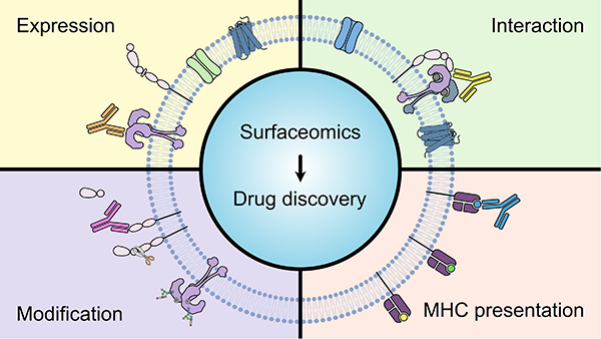

The cell surface proteome, or surfaceome, is the hub for cells to interact and communicate with the outside world. Many disease-associated changes are hard-wired within the surfaceome, yet approved drugs target less than 50 cell surface proteins. In the past decade, the proteomics community has made significant strides in developing new technologies tailored for studying the surfaceome in all its complexity. In this review, we first dive into the unique characteristics and functions of the surfaceome, emphasizing the necessity for specialized labeling, enrichment, and proteomic approaches. An overview of surfaceomics methods is provided, detailing techniques to measure changes in protein expression and how this leads to novel target discovery. Next, we highlight advances in proximity labeling proteomics (PLP), showcasing how various enzymatic and photoaffinity proximity labeling techniques can map protein–protein interactions and membrane protein complexes on the cell surface. We then review the role of extracellular post-translational modifications, focusing on cell surface glycosylation, proteolytic remodeling, and the secretome. Finally, we discuss methods for identifying tumor-specific peptide MHC complexes and how they have shaped therapeutic development. This emerging field of neo-protein epitopes is constantly evolving, where targets are identified at the proteome level and encompass defined disease-associated PTMs, complexes, and dysregulated cellular and tissue locations. Given the functional importance of the surfaceome for biology and therapy, we view surfaceomics as a critical piece of this quest for neo-epitope target discovery.

## 1 Introduction

### 1.1 The Cell Surface Proteome Is Critical for Biology and Medicine

The cell surface proteome, or surfaceome, is the hub for cells to interact with the outside world. In metazoans, it is broadly responsible for initiating cell signaling, metabolite transport, cell–cell interactions, and immune surveillance. The functional importance of the surfaceome is reflected in its tremendous genetic commitment to it: roughly a quarter of the human genome encodes membrane or secreted proteins.^1,2^ Recent proteomics data sets have validated that more than half of the surface proteins predicted from coding sequences do indeed appear at the cell surface across human cells.^1,3^ Surfaceomics studies are crucial to understanding this compartment beyond genetic annotation, as it is well-known that steady-state transcript and protein levels are not strongly correlated due to differences in synthesis rates, trafficking, and stability.^4^ Given the functional importance of the surfaceome and accessibility, it is no surprise that it is a major target for drug discovery. About half of small molecule drugs and virtually all protein therapeutics engage surface or extracellular proteins.^5^

Despite the functional importance of the surfaceome, we are only beginning to exploit potential drug opportunities. For example, protein therapeutics represent about half of the major revenues sold by pharmaceutical and biotech companies, yet there are currently less than 50 membrane or extracellular proteins targeted by approved drugs.^6^ There is a tremendous need to identify new drug targets in the surfaceome. In the past decade, the proteomics field has made significant strides to understand how surfaceome changes from health and disease in terms of proteins expressed and their levels,^7−9^ post-translation modifications,^10−14^ and dynamic and static protein complexes at high resolution that are formed.^15−19^

### 1.2 Basic Physical Differences between the Surfaceome and the Cytosol Mandate Different Proteomics Approaches

When studying the surfaceome, it is important to appreciate that the relative amount of protein in the surfaceome is about 100-fold lower than the total protein in the cytosol (Table 1). This can be gleaned from a simple calculation considering that the volume of the surfaceome (the “skin of the cell”) is about 100-fold smaller than the volume of the cytosol yet the protein density for both is about the same.^20−22^

**Table 1:** Basic Physical Features of Cytosol and Surface Proteomes

	Cytosol	Surface	References
Annotated number of encoded genes	∼14,000–17,500	∼3,000–6,500	SURFY, UniProt, SwissProt
Diameter/Thickness	cell diameter ∼ 20 μm (varies 10–100 μm)	∼0.03 μm (includes est. protein shell)	ref (20−22).
Volume	∼4 pL	∼0.04 pL	Calculated^a^
Relative amount of total protein	1	0.01	Calculated^a^
Protein copy #	∼100–100,000,000	∼10–1,000,000	ref (23−25).
Abundance range	10^6^	10^5^	ref (23−25).
Concentration range	∼0.001–1000 μM	∼0.0001–100 μM	Calculated^b^
Average distance between proteins	50–100 Å	50–100 Å	ref (26−28).; Calculated^c^
Degrees of Freedom	3 translational;	2 translational;	First principles
	3 rotational	1 rotational	
Relative association rate	1	22–33 times faster	ref (29).
Environment	Reducing; mostly-SH; low Ca^2+^	Oxidizing; mostly S–S; high Ca^2+^	
Dominant PTM types	100s; mostly reversible	10s; mostly irreversible	ref (30).

a Mammalian cells vary in size depending on type and stage of the cell cycle but average about 20 μm in diameter.^21,22^ Assuming a spherical shape this translates to a volume ∼ 4 pL. One can roughly estimate the volume of the surfaceome from the surface area of a 20 μm diameter cell (∼1.2e5 μm^2^) and the thickness of the surfaceome (assuming the lipid bilayer is ∼ 7.5–10 nm thick^22^ plus a distance ∼ 10 nm above and below for a reasonably sized membrane protein (∼250 kDa). This translates to a volume of ∼ 4 × 10^–2^ pL, or roughly 100-fold smaller volume than the cytosol. This, coupled with estimates that protein density is about the same in the membrane and cytosol (see below), means there are 100-fold lower amounts of protein in the cell membrane compared to the cytosol.

b Estimated from the volume of the cytosol the concentrations of proteins range about 6-log units, from about 1 nM (e.g., transcription factors) to 1000 mM (e.g., actin) with an average of about 50 nM.

c The density of proteins in the membrane is estimated to be about ∼ 30,000 proteins per mm.^226−228^ Taking the inverse ratio and adjusting units translates to an area of about 3,300 Å^2^ per protein; taking the square root of this predicts the distance between proteins of about 60Å.

Comprehensive quantitative proteomics studies show that cytosolic protein abundances range from 100s to 100s of millions of copies per cell.^23^ Although the same comprehensive proteomic analysis is lacking for membrane proteins, there have been copy number estimates for specific membrane proteins that range on the low end from 10s of copies per cell for peptide MHC complexes,^24^ up to several million copies per cell for highly expressed signaling molecules such as constitutively activated HER2.^25^ Based on the volume calculations above for the surfaceome, this would translate to concentrations varying about 5-log units, from about 0.4 nM to about 40 mM. Thus, proteins in the cytosol and surfaceome vary over similar abundance ranges and remarkably similar concentration ranges when corrected for volume. Even though the cytosolic proteome and surfaceome have similar concentration ranges, the fact that the surfaceome is 100-fold smaller means that the absolute abundance of surface proteins is, on average, 2 orders of magnitude lower than those in the cytosol. Thus, to get the deepest proteomic coverage, current surfaceomics methods are geared to enrich surface proteins over the vast excess of cytosolic proteins. Section 2 of this review provides an overview of current proteomics and nonproteomics methods of probing the surfaceome and a comparison of mainstream proteomics surfaceome enrichment strategy is detailed in Table 2

**Table 2:** Comparison of Surfaceomics Approaches

Method:	None; whole cell lysate	Membrane enrichment	Sulfo-biotinyation	CSC	WGA-HRP	FA-Ir PLP	Cholesterol-conjugated APEX
Chemistry:	none	none	Surface-amines	Aldehydes	Phenoxy-radicals	Carbene radicals	Phenoxy-radicals
Cell preparation:	Solubilization	Gradient/Isolation	None	Periodate	None	None	Cholesterol functionalization and sortase tethering
Specificity:	none	none	Lys	CHO	Tyr	None	Tyr
Typical sample size (number of adherent cells)	1 × 10^6^	1–10 × 10^6^	5–10 × 10^6^	5–10 × 10^6^	0.5 × 10^6^	2.5 × 10^6^	0.5–1 × 10^6^
Estimated total protein ID	12,000	2–3,000	2–3,000	2–3,000	2–3,000	1,000	350
Estimated membrane protein annotation	3%^a^	30%^55^	Up to 50%^85^	40–60%, up to 90%^10^	40–60%^68^	90%^17^	60%^64^
Reference for technology and recent application		ref (54, 55).	ref (56, 58).	ref (77, 84).	ref (68).	ref (17).	ref (64).

a Estimate based on analysis of whole cell lysate of K562 lysate data identifying 208 of 7076 proteins annotated in the SURFY database.

When considering proteomic approaches to understanding protein–protein interactomes on the cell surface, it is essential to appreciate the total protein density, how far proteins are from each other, and the range of affinities for molecules in complexes (Table 1). The density of membrane-associated proteins is estimated to be about ∼ 30,000 proteins per μm^2^,^26−28^ which translates to an average distance between proteins of about 60 Å (Table 1). This is striking as the diameter of an average single domain protein or growth factor (∼25 kDa) is about 30 Å, and the diameter of a common immunoglobulin such as a Fab arm of an antibody (∼50 kDa) is ∼ 60 Å. This means that proteins are so densely packed in the membrane that they invariably touch one another, especially when considering the lipid coats surrounding them. This estimate comports with other data that the mass of protein and lipid in the membrane are roughly equal, and that of the lipid is involved in mainly forming single-layer shells around proteins. Overall, the protein density in the membrane is not much different than the protein density in the cytosol (∼100 mg/mL).^31^ Crowding in the membrane and cytosol is known to slow diffusion rates in both compartments relative to an infinitely dilute state.^32−34^

A most significant fundamental difference between proteins in a two-dimensional membrane versus a three-dimensional cytosol is that they lose three of their six degrees of freedom: proteins can only translate in two dimensions in the plane of the membrane, and can only rotate along the *Z*-axis but not flip along the x- or *y*-axis without huge free energy consequences. The lipid bilayer is well-known to undergo a phase transition around 12–15 °C.^35,36^ Even in the liquid phase, it has been estimated that diffusion in the membrane is 10–100 fold slower than in the cytosol.^34^ However, the slower diffusion rate in the membrane is more than compensated by the reduced degrees of freedom. Recent studies using fluorescence correlation spectroscopy at the single molecule level show that molecules in a lipid bilayer can associate with partners 22 to 33-fold faster than a similar search in solution.^29^ On a macroscopic scale, this rate differential is even greater than that between flying and driving. In some sense, the fluidity of the membrane represents a high-speed cellular transit system that allows membrane proteins to rapidly rearrange and interreact unless they are anchored to the cytoskeleton or other structures (for review see ref (37))

The faster association rates for membrane proteins mean that for comparable occupancy as seen for cytosolic proteins, their intrinsic K_a_ values can be proportionately lower. The intrinsically lower affinity has implications for the evolution and design of membrane proteins as it is “easier” to evolve or design a stable protein assembly in the membrane; of course, this could come with a specificity penalty, too. This also has implications for studying complexes within the 2D membrane because when solubilized in 3D for typical affinity pull-downs, they would more rapidly dissociate. This highlights the importance of new proximity labeling proteomics (PLP) that allow photographic “snap-shots” for determining protein neighborhoods *in situ*, without the need to solubilize the surfaceome where these interactions could fall apart. The density considerations above would also strongly argue for high-resolution PLP approaches for defining first shell interactomes as opposed to low-resolution approaches that capture more broadly.

Another major difference between the inside and outside of the cell is that the outside is oxidizing, and the inside is reducing. Virtually all cysteines in extracellular proteins are paired in disulfides.^38^ Inside the cell, almost all cysteines are reduced. There are some notable exceptions to this rule; for example, serum albumin has a number of free cysteines, and virtually all of them are in reduced form.^39^ Salt and metal concentrations also differ inside versus outside.

Another fundamental difference between inside and outside the cell is the complexity of post-translational modifications (PTMs). Inside the cell, there are hundreds of types of PTM enzymes, and most PTMs inside are reversible like phosphorylation-dephosphorylation, ubiquitination-deubiquitination, methylation-demethylation, etc. In contrast, outside the cell, there are fewer than a dozen established PTM types, and all are essentially irreversible, such as glycosylation, sulfation, disulfide-bonds, glycosylphosphatidylinositol (GPI) anchoring, and proteolysis. Thus, once a protein emerges on the cell membrane through the secretory process, it is essentially done with processing except for regulated proteolysis, which is known for selective receptor activation, growth factor shedding, and matrix remodeling. There can also be some glycan remodeling postsecretion.

The surfaceome is a dynamic proteome that is constantly changing in response to cellular demand and engagement with the outside. Not only are proteins changing due to expression level and trafficking from the ER/Golgi secretion system, but the cell surface can exchange proteins with other organelles such as the lysosome or specialized vesicles involved in neurotransmitter, glucose, lipid, or cholesterol transport.^40^ All these features above make the surfaceome a fascinating yet challenging compartment for proteomics. The community has made significant progress in tailoring enrichment and labeling technologies to specifically study the surfaceome in all its complexity. This review will focus on new tools and approaches for the study of the surfaceome.

## 2 Overview of Surfaceomics Methods to Measure Changes in Protein Expression

### 2.1 Regulation of Protein Expression on Cell Surface and Surface Protein Topology

The cell surfaceome encompasses all cell surface proteins tethered to the extracellular leaflet of the plasma membrane, as well as a portion of secreted and shed proteins that may stay tightly associated with the cell. Surface protein synthesis typically begins at the ribosome, where the nascent signal peptide is recognized by the signal recognition particle (SRP) and trafficked to the SRP receptor on the endoplasmic reticulum (ER) membrane.^41,42^ In most cases, the signal peptide is then cleaved, and the nascent protein is cotranslocated into the ER lumen. A membrane protein, as opposed to a secreted protein, is anchored on the cell membrane using a transmembrane domain or, in some cases, by a lipid anchor, typically a glycophophatidylinosol (GPI) anchor. Finally, ER vesicles are shuttled to the Golgi apparatus, where luminal proteins are modified by glycosylation and presented on the extracellular leaflet of the plasma membrane (for review see ref (43)).

Surface proteins have four basic topologies.^44−46^ Single-pass transmembrane receptors represent about 70% of the membrane proteins and comprise two families: Type I (N-terminus out; ∼ 80%) and Type II (N-terminus in ∼ 15%).^46^ These often function as signaling receptors, adhesive molecules, and immune surveillance, either as antigen presentation or immune cell sensing. Extracellular-facing enzymes are also a part of this single-pass class; almost 300 of the 500 proteases encoded in the human genome have their active sites topologically outside.^47^ Examples of intracellular facing enzymes include membrane-bound receptor Tyr- or Ser/Thr kinases (∼70)^48−50^ or receptor-type protein tyrosine phosphatases (21 PTPRs)^51^ have their active sites inside and are generally regulated from the outside by engaging their ectodomains. Multipass receptors represent the rest of the surfaceome topologies (∼15%) and generally contain from 2 to 12 TM domains; these often function as small molecule sensors such as the 7-TM GPCR family or as nutrient, or electrolyte transporters.^46^ Beyond transmembrane anchored surface proteins, there is a small class of ∼ 150 GPI anchored proteins that function as binding sites or present ecto-enzymes such as NT5E (a 5′nucleotidase).^52^ It is important to note that the GPI anchors proteins by their C-termini on the extracellular leaflet of the plasma membrane, while other protein–lipid modifications, such as myristoylation or palmitoylation, often anchor cytoplasmic proteins on the inner leaflet of the plasma membrane or other organelles. Most membrane and secreted proteins have signal peptides that can be recognized by bioinformatics, but many do not, which further highlights the need for proteomics.

### 2.2 Proteomics Techniques to Detect and Quantify Surfaceome Abundance

To provide the deepest coverage of the surfaceome over cytosolic contaminants, most investigators use enrichment methods. In this section, we will review and discuss different techniques ranging from low-resolution (e.g., membrane fractionation) to high-resolution (e.g., membrane impermeable probes and glycoprotein enrichment) surfaceome enrichment. We will also briefly review antibody-based surfaceome technologies and conclude with a bioinformatic analysis of membrane proteomes.

#### 2.2.1 Membrane Localization/Fractionation

**Figure 1:**
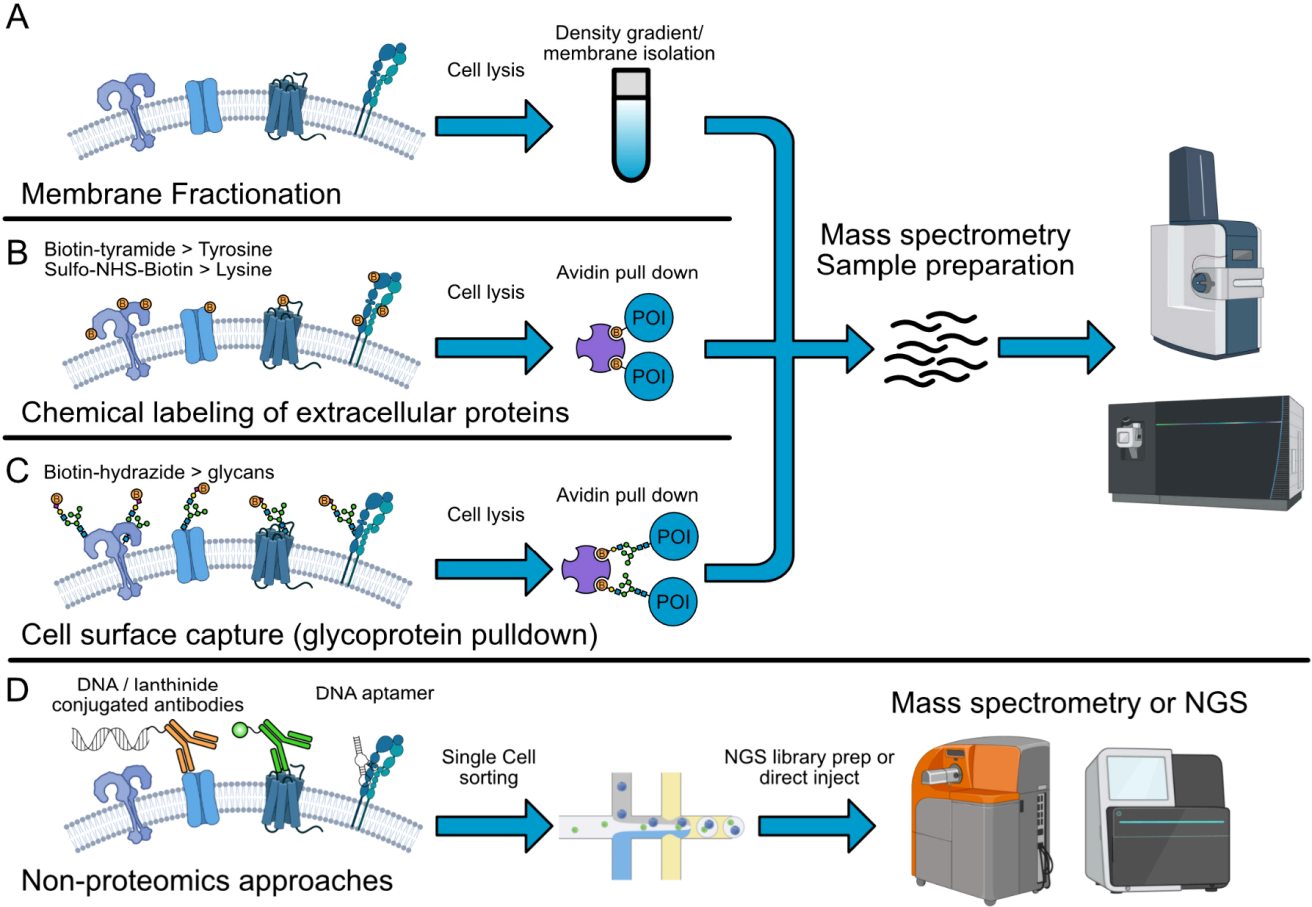
Schematic of cell surfaceome enrichment techniques. (A) Membrane fractionation or membrane isolation kits followed by mass spectrometry analysis (B) Chemical modification of cell surface proteins with impermeable probes such as sulfo-biotin-NHS or peroxidases. (C) Glycoprotein modification using with biotin-hydrazide. (D) Antibody-based methods such as CyTOF, MIBI, CITE-seq, DNA aptamer-based SomaScan and Phage-antibody NGS (PhaNGS). Portions of this figure were created with BioRender.com.

Perhaps the crudest enrichment method for the surfaceome relies simply on membrane fractionation (Figure 1A). This is typically done using a sucrose gradient where homogenized cell lysates can be separated using differential centrifugation in density gradients to isolate nuclear, cytosolic, mitochondrial, and mixed microsomal fractions combined of Golgi, endoplasmic reticulum, plasma membrane, and other vesicles.^53,54^ Alternatively, membrane proteins can be extracted by permeabilizing cells using mild detergent to release cytoplasmic proteins followed by a second detergent-based extraction of the remainder of membrane-bound proteins.^55^ These first-generation membrane isolation techniques have been a mainstay in the field and are especially useful in processing tissue samples typically analyzed by bespoke Western blots. However, these approaches are labor intensive and do not cleanly differentiate membrane proteins from organelles such as ER, Golgi, and lysosome. These methods also do not differentiate between proteins tethered on the inner and outer leaflets of membranes.

#### 2.2.2 Membrane Impermeable Probes and Membrane-Bound Enzymes to Target the Cell Surfaceome

To increase the accuracy of surface protein identification, techniques have been developed that rely on the physical impermeability property of cell membranes (Figure 1B). Sulfo-*N*-hydroxy-succinamide ester-biotin (sulfo-NHS-biotin) was developed as a cell-impermeable protein labeling probe that rapidly labels amines.^56−58^ This and related cleavable forms were initially popular because of their simplicity and commercial availability. More recently, chemical proteomics strategies such as global analysis of surface functionality (GASF) have been developed to map the reactive lysine residues exposed on the cell surface of living cells. Specifically, a chemical probe OPA–S-S-alkyne can efficiently and selectively label the solvent-exposed lysines on proteins. The resulting labeled surfaceome can be biotinylated via click chemistries and pulled-downed for analysis.^59^ However, all these chemical methods suffer from significant labeling of cytosolic proteins, presumably from a small amount of cell lysis, which significantly decreases the sensitivity of surfaceome detection.

An alternative approach to labeling the surfaceome is proximity labeling with cell membrane-tethered or impermeable enzymes. Engineered ascorbate peroxidase (APEX) can be tethered to the membrane with a genetically encoded transmembrane domain. Upon addition of H_2_O_2,_ and biotin-phenols these enzymes will produce biotin-phenoxyl radicals that covalently label proteins primarily at Tyr residues. Although labeling is biased to the cell surfacome the neutral phenoxy radicals have long half-lives in the ms range so can penetrate cells and lead to broad-scale labeling of both membrane and cytosolic proteins (>50%). APEX has been widely applied to probe mitochondrial compartments,^60^ and membrane-tethered HRP has been used in probing surfaceome of synaptic clefts in drosophila and mice.^61−63^ Similarly, the cell surface can be prefunctionalized with cholesterol lipid groups, followed by sortase-catalyzed conjugation with an improved APEX variant (APEX2) for cell surface protein labeling.^64^

Recently we have applied wheat germ agglutinin conjugated to horseradish peroxidase (WGA-HRP) as a general surfaceomic labeling tool. WGA-HRP has long been used for histochemical labeling of tissues for neuronal imaging.^65^ WGA-HRP binds to cell surface glycans, so it does not require cell engineering or chemical conjugation as typically done for APEX. Like APEX, HRP generates the broadly diffusing biotin-phenoxy radicals that is cell permeable and can label neighboring proteins, including those that are not glycosylated, up to 50% cytosolic protein contaminants. WGA-HRP labeling is not significantly hindered by variability in individual protein glycosylation status^66^ nor does it bias tryptic digest patterns as NHS-sulfo-biotin that labels lysines.^67^ Although the popular method of cell surface capture (CSC) (described below) allows for a higher percentage enrichment of surface vs cytosolic proteins when compared to cell-tethered WGA peroxidase, the WGA-HRP method requires 5-fold fewer cells for the same number of membrane protein identifications. This has significant advantages when samples are limited. For example, WGA-HRP was used to profile extracellular vesicles (EV) with limited quantities, whereas CSC was not sensitive enough.^68^

The engineered promiscuous biotin ligase (BioID, TurboID, and miniTurbo) has been developed as a proximity ligation technique to label and enrich subcellular compartments.^69,70^ While BioID and TurboID has the advantage over peroxidases of not requiring a toxic H_2_O_2_ as a substrate, the slower kinetics, limited extracellular ATP, longer half-life of the probe (minutes), and corresponding diffusion distance of the reactive biotin-AMP intermediate compared to peroxidases make it potentially less useful for surfaceomics work. However, recent studies using structurally defined DNA substrates have shown that BioID and TurboID have a bimodal labeling pattern: a small proportion that is very short-range (∼6 nm) proposed to represent direct contact labeling and a much larger portion of long-distance labeling reflecting diffusion of the long-lived biotin-AMP.^71^

#### 2.2.3 Targeting Glycosylation for Enrichment

Protein glycosylation is perhaps the most characteristic post-translational modification for extracellular proteins and is present on >85% of membrane proteins (Figure 1C). Typically, glycans are linked to serine or threonine residues (O-linked glycosylation) or to asparagine residues (N-linked glycosylation on the N-X-S/T sequence motif). Protein glycosylation, and in particular N-linked glycosylation, is prevalent in proteins destined for extracellular environments on the plasma membrane and secreted proteins, making it a ripe handle for specifically enriching the surfaceome.

##### 2.2.3.1 Metabolic Labeling of Glycans for Glycoprotein Enrichment

Metabolic labeling can be used to introduce functional groups on the cell surface, such as azide or alkynes, for bio-orthogonal biotin labeling. For example, metabolic chemical reporters (MCRs), such as Ac_4_ManNAz via Staudinger ligation, have enabled selective labeling of glycoproteins suitable for subsequent MS analysis.^72−74^ Unnatural sugar moieties, typically GlcNAc or GalNAc analogs functionalized with azide or alkyne handles for click chemistry, can be metabolically incorporated directly onto protein substrates. It should be noted that GlcNAc is also a major PTM inside cells so one needs to triage these cytosolic GlcNAc contaminants from surfaceome labeling. Metabolic labeling is not uniform across all cell lines and requires culturing which may alter cellular metabolism and potentially stress cells that could alter the cell surfaceome.

##### 2.2.3.2 Hydrazide/Amino-oxy Labeling for Glycoprotein Enrichment

Sodium periodate (NaIO_4_) oxidizes vicinal diols on glycans (especially terminal sialic acids) to form aldehydes. The aldehydes readily react with hydrazide, to form stable hydrazone linkages.^75^ The enrichment technique called cell surface capture (CSC) developed by Wollscheid, Aebersold, and co-workers, utilized this chemistry to capture cell surface proteins onto hydrazide beads.^76^ The captured glycoproteins were washed extensively and released using PNGase F, an amidase that cleaves between the innermost GlcNAc and the *N*-glycan-modified asparagine. Treatment with PNGase F leaves an aspartic acid scar that reflects protein deaminidation (+1 Da), enabling detection using high-resolution mass spectrometry. Subsequent modifications to the protocol by Wollscheid and co-workers make use of commercially available biocytin hydrazide to enable more efficient labeling followed by immobilization of glycoprotein on avidin-based resin.^77^ Various groups further optimized the sodium periodate treatment to be more tolerable with cell culture conditions and lower temperature to prevent membrane trafficking (1–2 mM NaIO_4_, PBS pH 6.0–6.5, at 4 °C for 15–20 min).^78−80^ The addition of aniline as a catalyst speeds up the formation of hydrazone linkage and further improves labeling efficiency.^78,80,81^ Aminooxy-biotin increased the yield of the conjugation product. To increase the depth of coverage, many groups now analyze both the tryptic digest from the beads as well as the PNGase F fraction. The CSC method is very popular. Recently, the Gundry group tallied >266 published studies in the past 20 years^82^ that use some variation of CSC.

It is estimated that about half the protein-coding genes are translated at some level in any given cell.^1,83^ Given there are about 4,000–6,000 membrane and secreted proteins, one would estimate there should be 2,000–3,000 membrane or secreted proteins from a given cell. Current optimized CSC methods will routinely identify ∼ 1,000 membrane proteins from ∼ 5 million mammalian cells (depending on cell size) so the limit of detection is not comprehensive at this sample size. To this end, it is important to compare the efficiency and quality of the enrichment methods (Table 2). About 10% of proteins identified using deep proteomic coverage of whole cell lysates are membrane proteins, but being ∼ 100-fold less abundant makes quantitative analysis challenging. Membrane fractionation enrichment methods increase this percentage to about 20%, and enzymatic labeling using tethered peroxidase increases the proportion of membrane proteins to between 40 and 60% of the total proteins.^10,68^ The PNGase F release of proteolytically digested glycopeptides remains the most stringent method of purification, reaching up to 95% surfaceome proteins.^10^ Recent surfaceome methodology using automatic sample handling can substantially reduce the input requirement of cells, reduce background contamination, and increase the coverage of the surfaceome.^79,84^

While the current state-of-the-art surfaceomic techniques rely on glycan-based enrichment or glycan tethering, it is becoming increasingly clear that several factors can affect what’s truly on the cell surface. In cancer, this could be a result of changes in glycosylation pattern, altered endosomal trafficking, rebinding of secreted protein, or ectopic display of intracellular proteins. We believe that other methods of labeling the cell surface may expand the surfaceome identifications and circumvent the dependence on the glycan handle. As new mass spectrometers are developed, sensitivity will undoubtedly increase. The method of choice for surfaceome enrichment will ultimately hinge on the combination of instrument sensitivity, availability of starting sample material, and enrichment method.

#### 2.2.4 Quantification of Cell Surface Proteins by Mass Spectrometry

Historically, robust comparative protein quantification used techniques such as stable isotopic labeling using amino acids in cell culture (SILAC)^86^ or isobaric tags using tandem mass tags (TMT)^87^ for binary or multiplexed quantitative comparisons, respectively. The development of label-free quantification^88^ with matched samples allows a facile approach to compare samples but is prone to missing values for low-abundance proteins. Absolute quantification has largely been limited to targeted proteomics (MRM, PRM), where known concentrations of a protein can be made using isotope labeling (for review see reference (89)). More recently, Data-Independent-Acquisition (DIA)^90^ and, in particular, DIA-PASEF^91^ have been developed where precursor ions are isolated into predefined isolation windows and fragmented, such that all fragmented ions in each window are then analyzed and quantified. When a spectral library of peptide fragments (using traditional Data-Dependent-Acquisition, DDA) is well constructed, it can be used to search DIA data and quantify even low-abundance proteins with high confidence. This emerging approach can identify and quantify 2,000–5,300 proteins from single-cell whole-cell lysate.^92,93^

To date, the Human Proteome Project (HPP) has experimental data for 18,407 (PE1 level evidence) of the 19,750 (predicted) proteins.^94^ Of the 18,407 proteins, 868 proteins remain undetected by means of mass spectrometry – most of which belong to the “plasma membrane” (GO:00005886) or “extracellular region” (GO:00005667). A recent deep proteomics sequencing preprint further shows that hydrophobic peptides spanning the transmembrane regions are underrepresented by mass spectrometry-based detection methods.^95^ Surfaceome enrichment approaches have certainly extended our ability to detect surface proteins and will play a critical role in comprehensively detecting cell surface proteins.

#### 2.2.5 Nonproteomics Based Technology of Profiling Surfaceome

In the past decade, antibody-based approaches to profile the surfaceome have emerged as a more bespoke surfaceome profiling technology (Figure 1D). It has largely been enabled by multiplexed antibody-based detection using mass spectrometry (cyTOF,^96^ MIBI^97^) or NGS sequencing readout (CITE-seq,^98^ CODEX,^99^ and PhaNGS^100^). When the antibody panels are well validated, these technologies can reliably profile upward of 100s of surfaceome targets in a single experiment. In addition to antibody-based surfaceome approaches, the multiplexed DNA aptamer-based proteomics approach (SOMAscan) is a promising technology that can detect thousands of proteins used to profile mostly soluble proteins in solutions such as patient serum.^101,102^

Despite the advances in developing these multiplexing technologies, caveats include the heterogeneity in quality and specificity of the binders used, their broad range of affinities, and the variable number of cell surface proteins presented on different cells. Antibodies differing in off rates for binding (*k*_off_) can change the final percentage bound antigens after a given number of washes. Many antibodies that bind to the naked target *in vitro* do not bind on cells either because the target may be engaged with other proteins (that will vary from cell type) or the target is in a different conformation. Lastly, the dynamic range of the detection instrumentation is a limiting factor when considering the 5-log span of abundance of membrane proteins. Nonetheless, when a panel of cell-binding antibodies is optimized, antibody-based approaches to surfaceome profile can provide a robust approach for target-directed surfaceome profiling.

### 2.3 Data Processing, Annotation, and Interpretation for the Surfaceome

**Figure 2:**
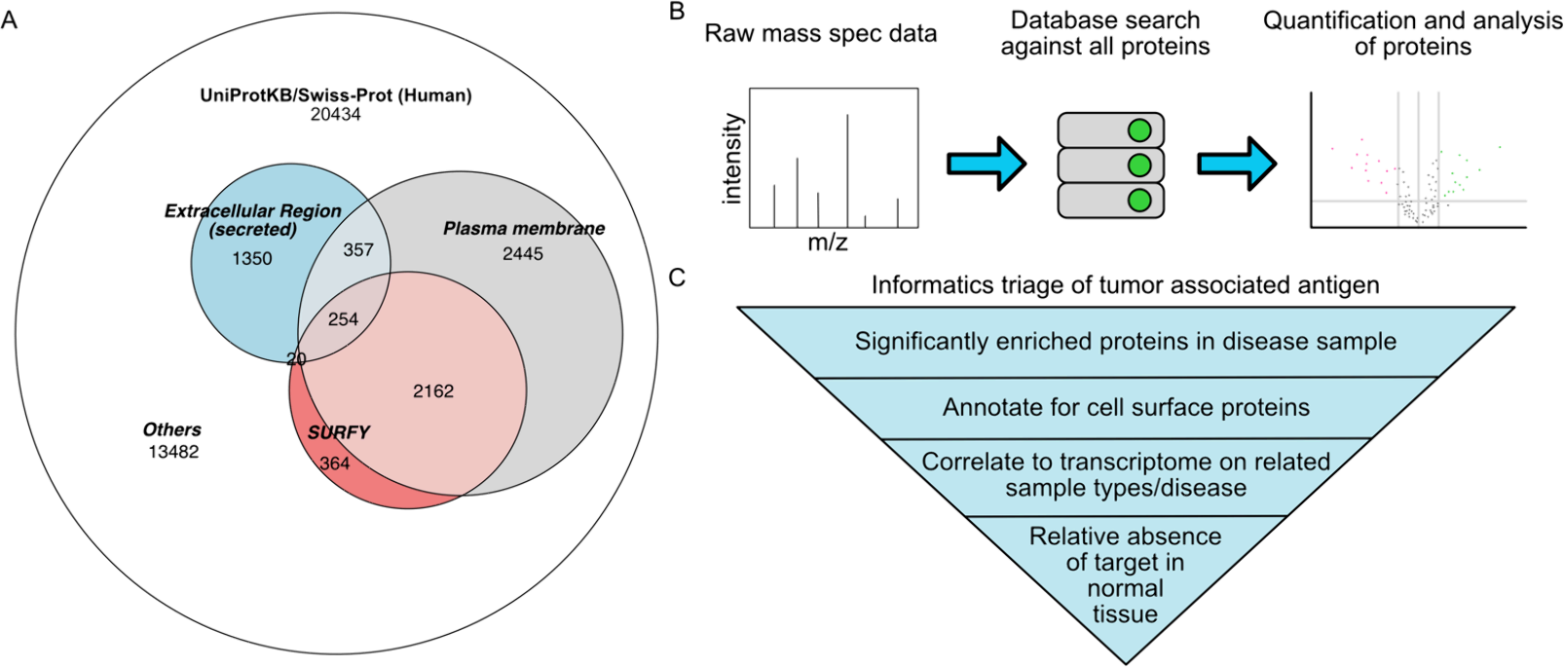
Annotation and analysis of surfaceome. (A) Annotation of surfaceome by Gene Ontology Cellular Compartment for plasma membrane (GO:0005886), extracellular region (GO:0005576), and SURFY^1^.^1^ (B) Typical data analysis pipeline of surfaceome. (C) Informatics triage for target discovery. Target triage begins with enriched proteins in diseased samples followed by annotation of cell surface proteins. Target candidates can then be cross referenced in existing database or transcriptomics data and further filtered for absence in normal tissues to maximize therapeutic index.

There is currently no consensus on a common list of proteins that constitute the ’surfaceome.’ In this section, we aim to shed light on why defining such a list is inherently complex and to provide novice surfaceome researchers with an understanding of the intricacies involved. There are ∼ 20,000 human protein entries in uniport (SWISSPROT) and annotation of membrane and secreted proteins rely mostly on signature motifs such as secretion signals and trans-membrane signatures. Various informatics used to localize surface proteins range from Gene Ontology for plasma membrane (GO:0005886, 6,019 proteins), machine learning trained on CSC data sets and prediction tools such as SURFY (2886),^1^ and tools that integrate experimental and prediction data to provide evidence-level of surface protein (VANEER, Figure 2A).^79,82^ It is also important to recognize that the surfaceome is dynamic and is well-known to exchange proteins with other compartments, such as the ER and Golgi, during biosynthesis (notably FTL3-ITD),^103^ endosomal recycling,^104^ signaling vesicles (notably neurotransmitter and glut-1 vesicles),^105^ lysosomal fusions during autophagy (notably LAMP1),^106^ and exosome biogenesis.^107^ While most proteins with signal sequences are secreted or remain in the membrane, some cytoplasmic proteins lacking a signal peptide can be noncanonically distributed onto the cell surface as detected by antibodies (notably heat shock proteins like HSP90, HSP70).^108^ Given the complexity of protein trafficking annotating the surfaceome in mass spectrometry searches should be done rigorously (Figure 2B). At a minimum, one should search against the entire Swiss-Prot database and report the percentage of proteins annotated as surface protein versus the total, including the percentage of cytosolic and background contaminating proteins identified (cross-referenced to databases such as the CRAPome^109^). We would also like to emphasize the value of depositing raw data as part of publication – this would enable researching by investigators and for future meta-analysis comparing different surfaceome methods.

From a surfaceome data set, a bioinformatics triage is typically performed such as that in Figure 2C. First, targets found to be significantly enriched in disease samples should be annotated and filtered for high-confidence surface proteins. One can correlate with transcriptomics data either experimentally or bioinformatically against broadly aggregated databases such as cBioPortal (cbioportal.org)^110^ or the Human Protein Atlas (proteinatlas.org). Lastly, to identify disease-associated target candidates the data can be cross-referenced for low expression in normal tissue determined experimentally using Western blot or against humans such as GTEx (gtexportal.org)^111^ or HubMAP’s Human Reference Atlas (humanatlas.io).^112^

In addition to identifying significantly enriched targets, pathway analysis can provide tremendous biological insight into the aggregated changes in the disease sample. In transcriptomics analysis, GeneSet Enrichment Analysis (GSEA) has been a mainstay in analyzing continuous data and assigning pathways that are dysregulated.^113^ This simple bioinformatics analysis is powerful and offers insight into the biology of a disease state beyond singleton target discovery. For example, GSEA identified dysregulation of glycosylation pathways in a panel of oncogene-driven isogenic cell lines that we would not have otherwise recognized using proteomics alone.^10^ This led to an in-depth glycoproteomics analysis and unraveling of highly differential glycosylation patterns in isogenic cell lines. Software has been developed for this type of analysis (PSEA-quant),^114^ and can also be performed by mapping protein ID to gene ID and performing traditional GSEA.^10^ This simple analysis enables the identification of dysregulated pathways from the surfaceome and augments target discovery pipelines.

### 2.4 Therapeutic Modalities against Cell Surface Proteins Are Dependent on Receptor Copy Number

**Figure 3:**
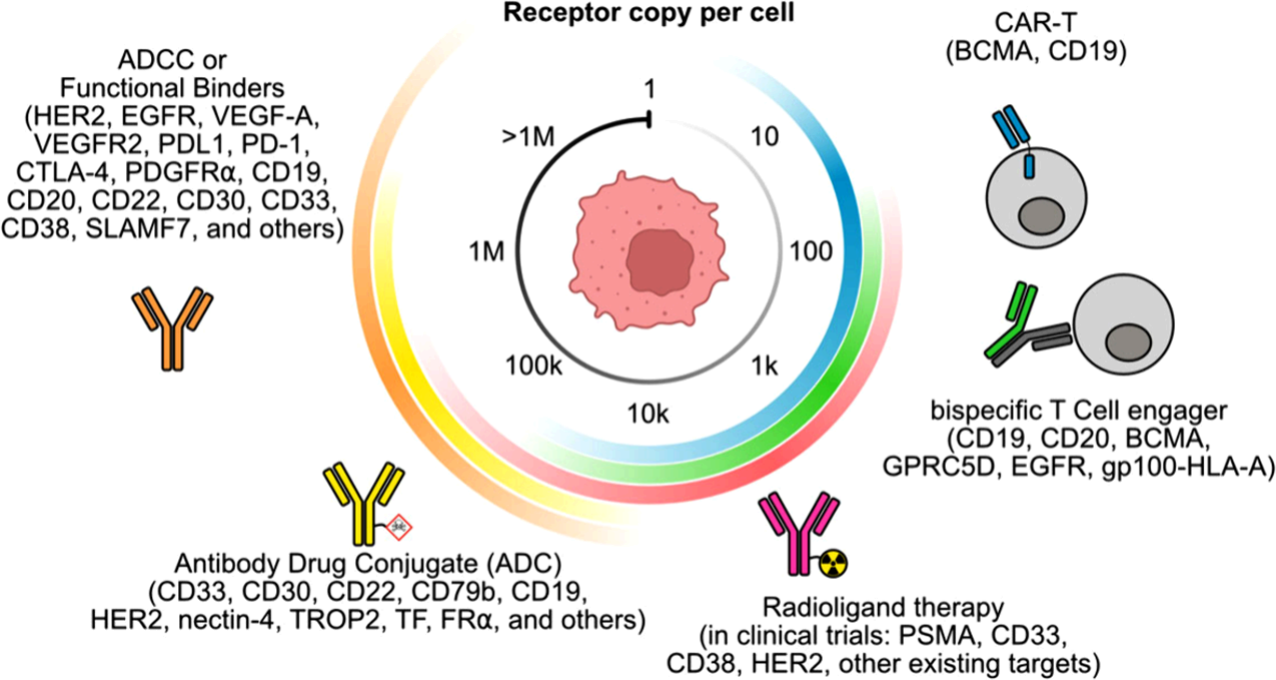
Efficacy of antibody modalities depends on the number of detectable receptor copies per cell. Clinically approved therapeutic targets for traditional IgG targets (ref (115).) for high abundance cell surface targets, followed by ADC (reviewed in ref (116).), radioligand therapy in clinical trials (ref (117).), T cell engager (ref (118).), and CAR-T (ref (119).).

For cancer target discovery, proteomics can help to determine the absolute target number, which guides the choice of antibody modality to use, and the target number relative to healthy tissue to establish the therapeutic index (Figure 3). For example, at a high receptor density of 100,000 – 1 million receptor copies per cell (e.g., HER2^120^ and CD20^121^), a native antibody that engages the immune system by antibody-directed cell cytotoxicity (ADCC) can be sufficient. Historically, most proteins targeted by cancer therapeutics are in this range due to their high abundance.^115^ At 10,000–100,000 copies per cell, an antibody-drug conjugate (ADC) is an attractive modality that brings in cytotoxic drugs,^116^ but it requires the receptor to be naturally internalized/recycled or the antibody itself to induce internalization.^122,123^ Below 10,000 receptor copies per cell, a bispecific T cell engager (TCE) or NK cell engager (NKCE) can provide greater killing with fewer binding events owing to avidity and cytotoxic potency when forming an immunological synapse.^118^ Currently, most TCEs bind CD3 and the victim cell through a tumor-associated antigen. The high end of T cell activation can lead to a toxic cytokine storm, thereby limiting the upper limit of therapeutic window. Below 1,000 receptor copies per cell, chimeric antigen receptor T cell (CAR-T) therapy is a promising modality as the signaling strength of the CAR can be tuned with enhanced potency for low antigen receptor density.^118,124^ An emerging modality is radioligand therapy (RLT).^117^ Similar to ADC, RLT employs antibodies appended with a radionuclide to elicit cytotoxicity, and it has the ability to target cancer with a broad range of receptors, ranging from millions of copies (in the case of HER2) to potentially 100s of receptors using α particle emitting radionuclides targeting a single receptor.^125^ New bispecific modalities such as extracellular targeted protein degradation (eTPD)^126^ and cell-targeted enzymes such as surface sialidase offer novel emerging technologies.^127^ Beyond antibody-based modalities, smaller formats from small molecules, peptide/cyclic peptides, and mini-binders such as knottins, have also been investigated clinically and may have the advantage of enhanced tumor penetrance.^128,129^

#### 2.4.2 Therapeutic Targets in Cancer

The use of surfaceomics holds great promise for the broad identification of surface proteins that change between health and disease states. Traditional bespoke biology studies comparing tumor and normal cells have historically led to transformational monotherapies such as trastuzumab (targeting HER2) in breast cancer and Rituximab (targeting CD20) in B-cell lymphoma.^121^ However, it is rare to identify novel singleton proteins in cancer with limited expression in normal tissue. Using unbiased surfaceome and bioinformatics approaches, several groups have identified panels of cell surface targets from models of oncogenic transformations,^80,130−132^ loss of tumor suppressors,^133^ drug-induced epigenetic changes,^78^ and chemotherapy resistance.^134^ Surface proteomics has helped to identify previously underappreciated cell surface targets such as CDCP1 and NT5E,^80^ ENPP1,^135^ CD72.^136^ Beyond target identification, a specific isoform (EGFRvIII),^137^ epitope (cleaved CDCP1),^138^ or conformation of a target that is differentially presented on a tumor (Integrin β2)^139^ compared to normal tissue substantially increases its selectivity.

Surfaceomics-based target discovery can detect the presence of a target, where even the most advanced informatics platforms cannot predict the fate of a cell surface target. Examples include protein retention in the ER in the case of FLT3 mutations in AML,^103^ protein shedding in case of mesothelin in ovarian cancer,^140^ MICA/B shedding in case of immune checkpoint inhibition,^141^ or Trop2/Epcam2 shedding in the case of lung cancer.^142^ There is also a class of under-studied targets whereby dysregulated protein homeostasis leads to ectopic expression of intracellular proteins such as HSP90. In particular, Haystead and co-workers showed that a noncell permeable small molecule conjugated to fluorophore or radioligand stained cells can target ectopically expressed cell surface HSP90 in a breast cancer model.^143^

To increase selectivity toward cancer targets, surfaceome discovery enables the identification of multiple tumor-associated antigen candidates that can be targeted using an “AND” gate approach. For example, Agard and co-workers at Genentech recently developed a dual targeting TCE against Ly6E and B7–H4 that is simultaneously expressed on approximately 50% of breast cancers, whereas normal tissue expression is limited and mostly orthogonal.^144^ Using this approach, the on-target cytotoxic of B7–H4 in normal tissue is largely mitigated, thereby expanding the therapeutic index. While Ly6E and B7–H4 are well-known targets of breast cancer, one can envision an integrated surfaceome target discovery informatics pipeline that identifies pairs of targets at similar expression levels restricted to the tumor of interest. Engineering a dual-antigen targeting antibody will not only circumvent the risk of on-target toxicity but also have the potential to mitigate resistance mechanisms where the target receptor is mutated or downregulated.

## 3 Profiling Cell Surface Neighborhoods

### 3.1 Recent Advances in Proximity Labeling Proteomics (PLP)

The surfaceome is a dynamic communication hub involving interactions among cell surface receptors, secreted proteins, hormones, adhesive molecules, and transporters that play crucial roles in signal transduction, cell–cell communication, and cellular nutrition. As described above, the two-dimensional nature of the membrane allows proteins to associate much faster and substantially reduces the intrinsic affinity needed to form specific complexes compared to cytosolic protein complexes.^29,34,145^ While affinity purification mass spectrometry (AP-MS) is often used to characterize stable 3D cytosolic complexes, AP-MS can easily miss transient and weak membrane complexes in 2D once they are solubilized in 3D lysates.^146−148^ The development of proximity labeling proteomics (PLP) that allows labeling of protein neighborhoods in situ, without membrane solubilization.

**Figure 4:**
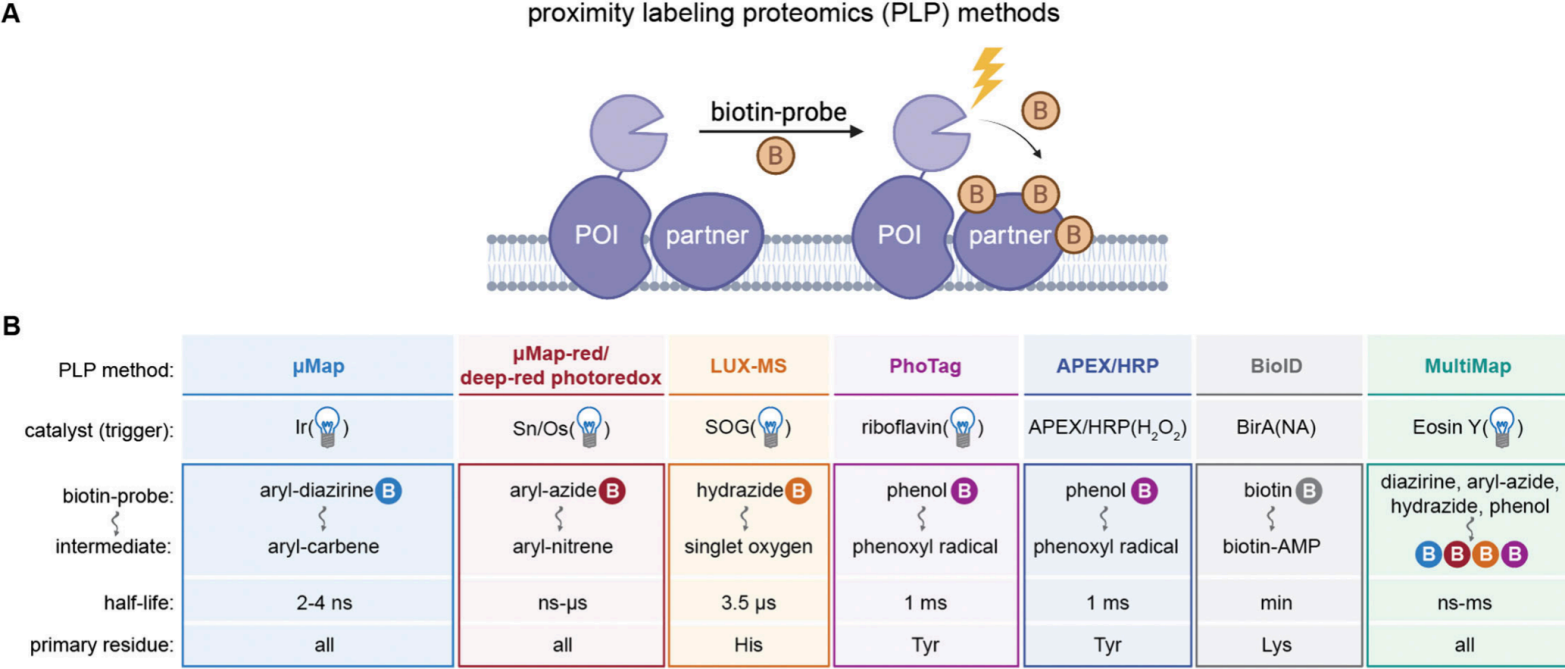
Overview of proximity labeling proteomics (PLP) methods. (A) General scheme for PLP where a catalyst is attached to the POI and partner proteins are labeled with a range-dependent biotin photoprobe. (B) Table describing the general features of PLP methods using iridium (μMap, ref (15, 17).), tin or osmium (μMap-red/deep-red photoredox, ref (156, 157).), singlet oxygen generator (LUX-MS, ref (158).), riboflavin (PhoTag, ref (159).), peroxidases (APEX/HRP, ref (160).), biotin ligases (BioID, ref (69, 161).), and Eosin Y (MultiMap ref (19).). Note that the labeling radius is tied to the reactivity (half-life) of the reactive intermediate, the leash distance from POI to photocatalyst, as well as whether residue exposure at the direct contact site can bias labeling.

An increasing number of PLP approaches have been introduced over the recent years (Figure 4). All PLP methods share some common features: an enzyme or photocatalyst is attached to the target protein of interest (POI). This catalyzes the activation and/or transfer of a soluble biotinylated probe to proximal proteins, forming a covalent bond between the probe and the target protein (Figure 4A).^149,150^ The various reactive species that researchers have used and their chemical features have been discussed in seminal reviews in the field.^151−492^ In the meantime, the nature of such approach can produce dramatically different outputs depending on the labeling distance, a result of both reactive intermediate half-life, its reactivity toward amino acid side-chains, and proximity to the target (Figure 4B). For this reason, it is critical to understand the maximal range and specificity of labeling and to have rigorous nonspecific controls to confidently define immediate, distal and nonspecific interactions in the crowded environment of the membrane. Given that PLP methods for intracellular proteins have been extensively reviewed,^149,150,152−155^ in this section, we will focus on reviewing PLP methods that can be used to map the interactions of cell surface proteins. We will discuss each method design, reported characterization efforts of the techniques, as well as applications to elucidate cell surface neighborhoods.

#### 3.1.1 Biotin Ligase and Other Enzymes for Proximity Labeling

##### 3.1.1.1 Biotin Ligase Generating Biotin-AMP

The pioneering PLP work of BioID utilizes biotin ligases exemplified by enzymes such as BirA*, and second-generation derivatives with faster kinetics, TurboID or miniTurbo. Upon the addition of biotin substrate and ATP, these enzymes catalyze the formation of a reactive biotin-AMP species that labels nearby Lys residues. In order to perform BioID on cell surface proteins, researchers have genetically fused the biotin ligases to the extracellular POI. After labeling, cells are lysed and enriched for biotinylated proteins using streptavidin or neutravidin beads. Enriched proteins are digested and identified by MS. First generation of biotin-ligase-based proximity labeling used a biotin ligase variant BirA* (R118G), which required a reaction time of more than 12 h for adequate accumulation of biotinylation.^69^ The catalytic efficiency of labeling was significantly increased with TurboID and miniTurbo, shortening the incubation time to 10 min which allows for probing events with faster kinetics.^162^ A recent study in 2024 from the Gingras lab demonstrated the possibility of performing TurboID on the cell surface.^163^ This extracellular TurboID, namely ecTurboID, identified an association between EGFR and low-density lipoprotein receptor (LDLR) upon EGF stimulation, which also changed the LDLR neighborhoods.

Since the first establishment of the BioID platform in 2012, the Roux group measured the extent of labeling in the intracellular setting using the nuclear lamina and the nuclear pore complex (NPC), whose diameters are 15–20 nm and 33–39 nm, respectively.^69,161^ Given that almost the entire NPC complex was labeled and identified in proteomics, which is not surprising given the established half-life for biotin-AMP hydrolysis (minutes in water),^164,165^ the authors reported a practical labeling radius of labeling for Biotin-AMP of ∼ 10 nm. This represents a minimum labeling radius in this particular setting. Recently, the Chen and Zou groups reported labeling patterns on both DNA origami scaffolds and rigid dsDNAs as fine nanorulers for distance measurement of labeling in vitro.^71^ By fixing BioID or TurboID to one single site, they found that labeling with BioID and TurboID peaked at 6–7 nm. Though they pointed out that biotin-AMP half-life at neutral pH should be minutes, this confined labeling radius is possibly due to contact-based mechanisms for BioID and TurboID. They additionally noted that contact-dependent labeling will be affected by steric accessibility of amino acids and thus may bias labeling efficiency. (Figure 4B). It is also noteworthy that the labeling radii could depend on cellular conditions. For example, free-radical quenching metabolites and enzymes could shorten the reactive half-lives and thus the labeling radii of these PLP probes. It is useful to establish labeling radii when conclusions depend upon it.

BioID and TurboID have also been appropriately used for long-distance labeling of proteins across cell compartments, including the nuclei, endosomes, mitochondria, ER, and plasma membrane. In 2016, the Sanyal group fused engineered BirA* onto ubiquitin-specific peptidase 12 (USP12), a key component of the T cell receptor complex on the cell surface.^166^ By performing proximity labeling in both resting and activated Jurkat cells, they found several new candidates, including LAT and TRAT1, and successfully confirmed their interactions with USP12 during TCR signaling through functional validation. In 2018, the Borner and Weissman groups used BioID to map the interactions of the ER membrane protein complex (EMC) and discovered multiple membrane proteins engaging EMC during their biosynthesis.^167^ The Gingras group has extensively used BioID for cytosolic interactomes and when combined with AP-MS provides higher confidence identification of dynamic cytosolic interactomes.^168−170^ More recently in 2023, the Kosako and Sawasaki groups demonstrated direct conjugation of AirID, an ancestral BirA, on antibodies or antibody Fab fragments to achieve cell surface target biotinylation.^171^ Structural modeling showed that the Fab-based strategy brought AirID closer to the target, thus providing enhanced efficiency compared to that from the antibody-based strategy. In another study in 2024, the Peeney group fused BioID and TurboID on matrisome protein TIMP2 to study the interactome of secreted factors.^172^

##### 3.1.1.2 MiniSOG Labeling Using Singlet Oxygen

MiniSOG, a small fluorescent flavoprotein, was originally developed for electron-microscopy studies by the Tsien and Shu groups.^173^ It can be genetically fused to target proteins and has also been used for PLP.^174^ This 15 kDa protein contains a flavin mononucleotide (FMN) cofactor and can generate singlet oxygen (^1^O_2_) allowing for labeling using phenol-biotin without the need for exogenously added toxic H_2_O_2_ used for peroxidases. In 2022, the Li group developed a photoactivation-dependent proximity labeling (PDPL) platform where POI is fused with the miniSOG protein.^175^ Upon blue light illumination, the enzyme can generate singlet oxygen from dissolved O_2_ to oxidize proximal histidine residues and allow subsequent covalent attachment of an amine-biotin probe for organelle-specific labeling. In parallel, the Zou group used miniSOG to establish a platform named RinID.^176^ By adding a higher concentration of amine probes, a shorter labeling time allows RinID to label protein interactions with higher spatiotemporal resolution including pulse-chase applications. While the maximal labeling radius using singlet oxygen has not been rigorously established, the half-life in water is about 3.5 μs in water,^177^ theoretically rendering a labeling radius between nitrene and difussive phenoxyl radicals (Figure 4B).

##### 3.1.1.3 LOV* Labeling via Singlet Oxygen and SET

Light-oxygen-voltage (LOV) domains are also FMN-binding proteins. In addition to its wide application in optogenetics and neuroscience, these <19 kDa proteins can also generate ^1^O_2_ in cells. In 2023, the Muir group established a platform named light-induced interactome tagging (LITag) by using an engineered LOV domain, LOV* and phenol-biotin.^178^ By showcasing this technique with PARP1 and MVP neighborhoods, they demonstrated rapid labeling by LITag that can be utilized for time-resolved studies. In this work, they also investigated the mechanism behind LOV*-induced labeling and proved that both single electron transfer (SET) and singlet oxygen generation mechanisms are operational in this system.

##### 3.1.1.4 PUP-IT Labeling with PafA Substrate

In 2014, Zhuang, Wells and co-workers developed the NEDDylator, which allowed for transfer of the ubiquitin homologue NEDD8 to E3 Ligase substrates that it contacted.^179^ This was later applied to label the binding partners of dasatinib.^180^ With the goal of mapping membrane protein interactions, in 2018, the Zhuang lab introduced a new proximity labeling system named PUP-IT by hijacking the bacterial Pup ligase, PafA. Fused to the cell surface POI, such as CD28 in mammalian cells, PafA activates exogenously added substrate with biotin and labels proteins in nucleophilic proximity.^181^ They demonstrated the platform on CD28 and identified two new membrane protein targets, RNF13 and MUL1, as potential interactors. More recently, they used PUP-IT to map the spatiotemporal interactions of MARCH5 during its translocation through mitochondria and peroxisome, which further demonstrated the wide applicability of this platform for intracellular labeling.^182^ The group also demonstrated a new generation labeler, PUP-IT2, where the Pup ligase PafA was downsized from 55 kDa to 7kD, allowing higher resolution for organelle-specific mapping.^183^ The advantage of the PUP-IT and NEDDylator platforms is that they require direct transfer of the PUP or NEED8, respectively, from the POI to protein neighbors thus providing unequivocal evidence that the two partners contact each other. This stands in contrast to the some other PLP methods that generate diffusible reactive intermediates. The potential limitation of PUP-IT is that it requires genetic engineering of cells, and it is possible that spatial constraints of the gene fusion may miss interactors even in direct contact. Reactions with PUP-IT and PUP-IT2 are typically done over 12 h incubation, thus limiting usage for time-resolved mapping.

##### 3.1.1.5 Tyrosinase Labeling

Tyrosinase is a bacterial enzyme that can oxidize substrates such as phenol to ortho-quinones and label proximal protein nucleophiles.^184^ In 2024, the Hamachi and Zhu group introduced tyrosinase to the POI and performed proximity labeling in mouse brain.^185^ In this work, they conjugated tyrosinase to neurotransmitter receptor ligands FITM and NAPS and injected the construct to the mouse brain for in vivo labeling. They found known interactors at specific neuron synapses from proteomics study and validated further by imaging. More recently, the Qin lab established TyroID where recombinant tyrosinase was fused with a HER2-specific nanobody, which allowed cell surface labeling.^186^ It is noteworthy that since tyrosinase labeling radius is expected to be higher than that of phenoxy radicals, the authors designed an alkyne-based membrane-impermeable phenol probe, termed AxxP in this workflow, which can be more compatible with the tandem TransitID workflow.^187^

##### 3.1.1.6 Other Contact-Based Reactions

In addition to PUP-IT and tyrosinase, other contact-based reactions or enzymatic processes have shown promising potential in mapping protein interactions engaged in cell–cell communication.^188^ Pioneering platforms using an engineered sortase were demonstrated in the workflows named labeling immune partnerships by sortagging intercellular contacts (LIPSTIC) and LIPSTIC2 from the Victora group,^189,190^ as well as enzyme-mediated proximity cell labeling (EXCELL) by the Peng Chen group. Alternatively, interaction-dependent fucosyl-biotinylation (FucoID) developed by the Li and Wu groups employed the fucosyltransferase and its substrate derivative with a biotin tag to achieve proximal labeling of proteins or cells.^191,192^ The Xing Chen lab also demonstrated endogenous enzymes can be used to activate quinone methide (QM)-based labeling to label interacting cells.^193^ These studies have focused mainly on cell level labeling. With proteomics analysis, these platforms can potentially help map protein–protein interactions as well.

#### 3.1.2 Engineered Peroxidase for Enzymatic Proximity Labeling

##### 3.1.2.1 Peroxidase Labeling with Phenol-Biotin (SPPLAT)

As described above, peroxidases produce phenoxyl radicals from phenol-biotin in the presence of H_2_O_2_ and extensively used for PLP. Horseradish peroxidase (HRP) is one of the most widely used enzymes for immunoassays, given its high stability, broad substrate specificity, high sensitivity, and commercial availability as an antibody conjugate. Peroxidase-induced proximity labeling can also be achieved by attaching HRP to a target protein using primary or secondary antibodies, which can then activate phenol-biotin in proximity and label proteins within ∼ 300 nm. In 2014, the Perrett and Jackson laboratories developed a platform called selective proteomic proximity labeling using tyramide (SPPLAT) to study the interactions of B cell receptor (BCR) cluster in chicken B cells DT40 and identified a chicken B-lymphocyte allotypic marker chB6 that interacts with BCR following receptor clustering.^194^

##### 3.1.2.2 HRP Labeling with EMARS

HRP can also be used to trigger reactions such as enzyme-mediated activation of radical sources (EMARS). In 2008, the Kotani and Honke laboratories developed an EMARS platform employing a biotin probe containing both aryl-azide and phenol to identify protein interactions in situ. The initial work used an HRP-labeled antibody to map the interactome of β1 integrin on the cell surface.^195^ By performing immunoelectron microscopy, they anchored HRP on a nickel grid and measured the distance of gold particles biotinylated after EMARS reaction. They observed 76% of particles within 200 nm from the anchored HRP and 94% within 300 nm, suggesting that the labeling radius is in the range of 200–300 nm. They also discovered known interacting proteins, such as EGFR, within a distance from the target protein. By further applying the method to TCR complexes, they further demonstrated the feasibility of the EMARS platform for cell surface interactomic mapping. In 2011, by combining EMARS with the antibody array system, the Kotani lab identified the β1 integrin interaction with RTKs such as EGFR and ERBB4, as well as CD20 interaction with fibroblast growth factor 3 (FGFR3) upon rituximab stimulation.^196^ More recently, the Kotani lab applied EMARS using a phenol probe to map the interactions of the SARS-CoV-2 spike protein.^197^ They found host proteins DPP4, Cadherin 17, and CD133 potentially interacting with the SARS-CoV-2 spike protein during viral-host infection. In a separate work, the group demonstrated the feasibility of mapping interactions on extracellular vesicles (EVs) *in vivo*, further expanding the generalizability of EMARS in various applications.^198^

##### 3.1.2.3 APEX Labeling with Phenol-Biotin

Engineered ascorbate peroxidases pioneered by the Ting group, such as APEX and APEX2, have become one of the most popular choices for proximity labeling.^160^ The series of APEX platforms achieves peroxidase-induced proximity labeling by genetically fusing APEX to POI.^60^ Upon addition of H_2_O_2_ and phenol-biotin, APEX efficiently generates phenoxyl radicals that predominantly label Tyr and much less frequently Trp, His and Cys.^149^ With its highly streamlined workflow, APEX has been widely used in protein interactome studies intracellularly, with recent efforts expanding to extracellular targets. In 2016, the Ting lab applied an optimized APEX workflow to map the interactions at the neuronal synaptic cleft.^61^ Instead of the traditional APEX or APEX2, they used HRP paired with a derivatized phenol-biotin with a long and polar polyamide linker (BxxP) for enhanced specificity for cell surface labeling. In 2018, the Wesche lab expressed a fibroblast growth factor 1 (FGF1) fusion protein with APEX2,^199^ Upon binding to FGF receptor on the cell surface, FGF1-APEX2 could catalyze labeling on the neighbors, capturing novel interactors including CSPG3 and CD44. In order to subtract nonspecific bystander labeling, they included wild-type U2OS that did not express FGF receptor as a control. In 2022, the Sorkin and Gygi laboratories fused APEX2 to the intracellular domain of EGFR and monitored neighbors upon ligand activation.^200^ By performing labeling at different time points, they kinetically tracked the enrichment of each protein target. Apart from known interactors, they found a protein located at the ER, Trk-fused gene (TFG), that regulates the endosomal sorting of EGFR. In 2023, the Blacklow and Kalocsay groups used a similar approach fusing APEX2 to the intracellular domain of Notch2 to capture the interactome of Notch2 during signaling. By tracking the location at different time points upon stimulation, several key regulators were found that bind with Notch2 upon JAG1 ligand binding, proteolysis and internalization.^201^ More recently in 2024, the Kruse, Blacklow, Kalocsay, and Susa groups adapted a similar workflow on the intracellular domain of CD19, a cell surface B cell receptor and profiled the known and novel regulators upon B cell activation with time resolution.^202^. APEX can also be conjugated to a ligand for cell surface labeling. In 2024, the Zhuang and Shui laboratories studied the signaling interactome of the GLP-1 receptor in pancreatic β cells and neuronal cells by conjugating APEX to its agonist GLP-1 peptide.^203^ Given that the GLP-1-APEX was exogenously added, no additional cellular engineering is needed. These examples demonstrate that APEX can provide functional neighborhoods with spatiotemporal resolution. When interpreting the resolution of labeling, it is critical to know the maximal labeling radii. The phenoxy radical has a long half-life of ∼ 1 ms,^151^ and maximal labeling radii estimated to be around 300 nm by single particle STED microscopy in aqueous solution.^18^ This large radius of labeling is very appropriate for validating cell–cell, or organellular interactions. For identifying immediate neighborhoods (10–30 nm), however, the long labeling radius will include false-positives due to bystander labeling in crowded cellular environments. APEX also requires cell engineering and exposes cells to mildly toxic H_2_O_2_ like other peroxidase labeling methods. The Ting lab tackled this issue of H_2_O_2_ toxicity by reporting a more recently developed LaccID.^204^ In this work, they engineered laccase that oxidases phenol using O_2_ instead of H_2_O_2_. By fusing the engineered enzyme to either a T cell receptor or chimeric antigen receptor against a tumor antigen NY-ESO-1, cell surface interactomics was achieved in a H_2_O_2_-free manner. A new study from the Martell lab also introduced an engineered version of APEX, named APOX, and demonstrated that the kinetics using the nitrophenol probe is greatly enhanced after directed evolution.^493^

When interpreting the resolution of labeling by APEX, it is critical to consider the labeling mechanisms and labeling environment. The phenoxy radical has a long half-life of about 1 ms, and the MacMillan group has recently estimated the maximal diffusive labeling radii in vitro to be around 300 nm by single particle STED microscopy.^18^ When measuring the distance for BioID/TurboID, the Zou and Chen groups also estimated APEX2 labeling distances on DNA origami scaffolds and dsDNA.^71^ While the half-life of phenol radicals is considered at ∼ 1 ms, they observed a dual mechanism where significant labeling with APEX2 at 7–12 nm is due to contact-dependent labeling and the distal labeling signal beyond 55 nm (or at ∼ 270 nm radius observed by STED microscopy)^18^ is potentially contributed by diffusive labeling of phenol radicals. Given the significant amount of diffusive labeling, one would expect that long-range and random diffusive labeling may be corrected with appropriate and necessary nonspecific controls. Radical quenchers may preferentially decrease the long-range labeling supporting this dual mechanism hypothesis.^205^ These studies highlight that enzyme-based proximity approaches can have a significant amount of contract-dependent labeling even when the probe is diffusible.

#### 3.1.3 Photoaffinity Labeling (PAL)-Based Proximity Labeling

Over the past decades, photoaffinity labeling has provided a unique opportunity to capture interactions in situ in an unbiased manner.^206^ In the 1970s the Knowles lab and others found a series of photoactivatable warheads including benzophenones and highly reactive diazirine warheads that when placed on ligands were useful for photoaffinity labeling (PAL).^207^ Photoactivation allows rapid time-resolved control that provides unbiased labeling of binding partners. PAL has emerged as an important technology for profiling interacting proteins. PAL relies on the selective anchoring of the PAL warheads directly to the POI by chemical/genetic means, or by using selective ligand or recognition motifs.

In early applications, researchers were able to use genetic code expansion to introduce PAL groups onto proteins selectively. By replacing amino acids with noncanonical amino acids that contain diazirine groups such as PhotoMet or PhotoLeu, the Thiele group successfully demonstrated metabolic incorporation of PAL groups.^208^ More recently, the Li group metabolically incorporated 2-amino-5-diazirinylnonynoic acid (PhotoANA), a bifunctional noncanonical amino acid that contains both a diazirine and alkyne tag, to profile the protein interactions between *S. enterica* and its host during infection.^209^ At the same time, the Kohler lab demonstrated the incorporation of diazirine-modified ManNAz, ManNDAz, onto cell surface glycans.^210^ By monitoring interactions using sialic acid biosynthesis, they identified CD22 and podocalyxin that can interact with sialic acids which is associated with cancer progression. Selective introduction of PAL groups to the POI can also be achieved through genetic code expansion technique originally pioneered by the Schultz group.^211−213^ By incorporating PAL groups on noncanonical amino acids, researchers have been able to anchor reactive warheads to the cell surface. In 2016, the Chen lab introduced a noncanonical amino acid PABK that contains an aryl-azide moiety to substitute Lys on EGFR.^214^ Upon UV activation, these anchored moieties can enable biorthogonal labeling and photo-cross-linking on the cell surface. Over the years, many different PAL groups have also been found compatible with the genetic code expansion technique, thus enhancing the chemical versatility of proximity labeling tools in live cells.^215^ In these cases, the labeling distances are very short. The reactive warhead cannot diffuse freely assuming the off-rate of the partners is higher than the half-life of the reactive intermediate. In this case, labeling radii are solely dependent on reactivity and the leash length of the warhead. While many have used native amino acids as a control, it is challenging to design controls with the same warhead at the same location for proteomics studies. Additionally, since PAL groups may be quenched by water, protein labeling signal is limited by the labeling efficiency and local PAL group concentration. Even though the diffusive distance is highly constrained, the yield of photoincorporation varies typically from 10 to 70% depending on the warhead and binding site.^216−218^

#### 3.1.4 Photocatalytic Proximity Labeling Proteomics (PLP)

Recently, there has been a surge of applications for light-activated photocatalysis reactions for PLP. Researchers have attached a variety of photocatalysts or photosensitizers to the POI to catalytically activate nearby soluble photoreactive probes for interactome profiling.

##### 3.1.4.1 Metal-Based PLP

Metal-based photocatalysts have been widely used in organic photocatalysis and have the advantage of high stability and high catalytic efficiency.^219^ In 2020, the MacMillan, Oslund and Fadeyi groups pioneered using an iridium (Ir) photocatalyst to activate diazirine-biotin and named this platform microenvironment mapping (μMap).^15^ Blue light (420 nm) can activate the Ir-catalyst and sensitize conversion of the diazirine to highly reactive carbene via a Dexter Energy Transfer (DET) mechanism. The Ir-catalyst was conjugated to a secondary antibody to recognize target-specific primary antibodies bound to the POI on the cell surface. They successfully demonstrated the platform on T cell surfacome by identifying targets including CD45, CD29 and CD47 on Jurkat cells, as well as PD-L1 at the T cell/victim cell synapse. This work demonstrated the feasibility of PLP to map interactomes on the cell surface. In 2022, the Oslund and Fadeyi group at Merck (now at InduPro) applied μMap to study the EGFR and c-MET interactomes on the cell surface. They used the same secondary antibody strategy and found known and new interactors of these receptor tyrosine kinases.^16^

Since the first successful demonstration of the Ir-photocatalyst, several new metal-based photocatalysts, as well as new reactive warheads, have been explored for their photocatalytic activity. In 2022, the Peng Chen group used an Ir-catalyst to activate caged quinone methide (QM) for cell surface protein PLP, which has been more adapted for mitochondria-specific labeling.^220,221^ In this platform named Cat-Ex, they anchored the Ir-catalyst to a HER2-specific affibody, and selective PD-L1 antibody Avelumab to map their local cell interactome.^222^ In the same year, the MacMillan group presented μMap-red.^156^ In this work, they conjugated a tin (Sn) photocatalyst onto antibodies and used red light to activate aryl-azide-biotin probes for PLP. They successfully demonstrated more than 5-fold greater tissue penetrance compared to blue light activation and applied it to profile erythrocytes from whole blood samples, indicating its potential application in complicated tissue samples. Red-light activation was also achieved by the Rovis, Oslund and Fadeyi groups by using osmium (Os) photocatalysts to activate fluorinated aryl-azide-biotin probes.^157^ By conjugating the Os-photocatalyst on antibodies, they demonstrated PLP on a cell-surface epithelial cell adhesion molecule (EpCAM). More recently in 2024, two additional studies demonstrated the possibility of using new photoprobes for near-infrared proximity labeling. The Shah and Rovis groups demonstrated that Os-photocatalysts can be triggered by red light to activate diazo labeling directly,^223^ which is a reactive species commonly considered to be generated during photoisomerization of diazirine.^224^ Further work from the Oslund and Fadeyi groups demonstrated that near-infrared illumination can trigger photocatalysis with photocatalysts, TTMAPP and *n*-Pr-DMQA^+^ to activate iodoperfluoroalkyl-biotin substrate and generate fluoroalkyl radicals for proximity labeling.^225^ By introducing a photocatalyst-conjugated CD45 antibody, iodoperfluoroalkyl-biotin probe and ascorbic acid, they compared biotinylated proteins with or without 30 min of red light and performed cell surface labeling both on cells and in tissue samples.

In applying PLP, it is crucial to understand the labeling radii of the photoprobes used. In 2022, the MacMillan group reported an elegant study examining the labeling radius of diazirine and phenol-based photoprobes using super-resolution microscopy.^18^ This work compared the labeling radii from the Ir-catalyst/diazirine-biotin pair to peroxidase/phenol-biotin pair. In their experiments, the Ir-catalyst and peroxidase were anchored to a secondary antibody which bound to the primary antibody to the POI on the cell target or by single molecule tethering to glass slides. By measuring labeling using stimulated emission depletion (STED) microscopy, they demonstrated that the Ir-catalyst, conjugated to 6–10 lysine sites on a secondary antibody, labeled proteins to a maximum range of 54 ± 12 nm compared to peroxidase-based proximity labeling at 269 ± 41 nm. The higher resolution for using the diazirine-biotin photoprobe is expected given it shorter half-life but one would expect even higher resolution given the relative difference in half-life to the phenol photoprobe of 100,000-fold (Figure 4). It is noteworthy that in this study, labeling radius are reflective to the measurement of labeling in an extracellular setting in aqueous solution which may vary due to environmental conditions. In 2024, we tested if the labeling radius for the Ir-photocatalyst could be further enhanced by direct attachment of the Ir-catalyst to the primary antibody and confining labeling at a single site versus 6–10 sites on the secondary antibody.^17^ We utilized site-specific labeling of methionines to attach the Ir-catalyst to 8 different single locations with a leash length of 3 nm onto a HER2 binding Fab derived from trastuzumab. The attachment was shown not to affect binding to the HER2 ectodomain. We observed broad labeling on all types of amino acid residues with the maximal labeling distance of around 11 nm from the site of Ir-photocatalyst attachment in the trastuzumab-HER2 complex. This maximal distance for carbene labeling was consistent with labeling sites in the structure of the known trastuzumab-HER2 complex, considering the 3 nm leash length for the Ir-catalyst from the conjugation site, the DET orbital overlap distance, and the diffusion expected over the 2–4 ns half-life. Applying these same considerations in a secondary antibody strategy predicted a maximal labeling radius of ∼ 50 nm, consistent with the reported labeling radius.^58^

Given the dense packing of proteins on the cell surface with an average protein distance at 6 nm, it is critical to quantify antibody-directed labeling to nonspecific labeling in order to best eliminate by-stander false positives. In the case of μMap, this is performed by simply omitting the primary antibody and labeling the cell surface with the non-interacting secondary antibody in the solution.^219^ This design reduces nonspecific labeling and is used in calculating fold-enrichment volcano plots to identify those proteins that are in close proximity to the photocatalyst. We found it useful to create a nonspecific membrane-tethered photocatalyst that sits directly on the membrane like the specific antibody-bound catalyst as opposed to nonspecific labeling from the solution.^17^ In fact, we observed higher resolution and enhanced enrichment specificity when combining site-specific catalyst attachment and normalizing to membrane-bound nonspecific control in our analysis.

In addition to antibody-assisted targeting strategies, other biomolecules, and chemical biology designs can be harnessed to introduce photocatalysts to the protein of interest. In 2015, the Nakamura lab introduced a ruthenium (Ru) photocatalyst to a small-molecule EGFR ligand Gefitinib.^226^ By illuminating with blue light, the Ru photocatalyst can generate singlet oxygen (^1^O_2_) and oxidize proximal His, Met, or Trp residues, allowing subsequent labeling with tyrosyl radical trapper reagents such as *N*′-acyl-*N*,*N*-dimethyl phenylenediamine. Through this mechanism, they achieved both EGFR-specific labeling in cells and oxidative protein knockdown via chromophore-assisted light inactivation similar to work from the Kodadek group.^227,494^ More recently, the Ru photocatalyst was used by the Li group to generate singlet oxygen at cell–cell synapses.^228^ With His residues oxidized with singlet oxygen, hydrazide can label the oxidized His and the interacting cells were tagged, allowing the identification of specific antigen presentation patterns between T cells and antigen-presenting cells. A more recent work from the Martell group used Ru photocatalyst to track proteins at the intercellular contacts.^229^ By introducing complementary DNA strands on two cells, photocatalyst become activated only in the presence of both cells, essentially generating an AND logic gate for labeling.

Similar small-molecule anchored designs have been used for intracellular studies including BRD4-binding JQ1, Src/Abl kinase inhibitor dasatinib and GPCR binders.^230^ In 2022, the MacMillan lab further demonstrated that the Ir-photocatalyst can be conjugated to azide-containing ManNAz, which were metabolically incorporated onto cell surface glycans.^231^ In this GlycoMap platform, the Ir-photocatalyst was incorporated using click reaction onto sialylated glycans, enabling subsequent nonspecific photocatalytic PLP. In 2023, the Muir and MacMillan groups demonstrated an alternative strategy to incorporate the Ir-photocatalyst using the split-intein technique. In this work, they successfully introduced the photocatalyst to nuclei and performed chromatin interactome profiling intracellularly.^151^ This technique was further expanded to profile intracellular interactions during stree granule assembly,^232^ phagocytosis,^233^ as well as shown to label RNA simultaneously.^234^

##### 3.1.4.2 Dye-Based Photocatalytic Proximity Labeling

While metal photocatalysts usually have high biological orthogonality in live cells, organic dye-based photocatalysts and photosensitizers have the unique advantages of facile synthesis and high chemical versatility while providing high catalytic efficiency.^235^ More importantly, many of these dye catalysts are readily commercially available, providing an alternative to metal-based photocatalysts that require up to 10-step syntheses.^15,157,236^

In 2021, the Wollscheid group discovered that some photosensitizers, including thio-rhodamine, can directly function as a singlet oxygen generator (SOG) for photocatalytic PLP in their LUX-MS platform.^158^ Similar to the miniSOG design, they used light to activate the photosensitizer and then added hydrazide-biotin to capture the oxidized residues, primarily oxo-His. In their study, the thio-rhodamine SOG photosensitizer was incorporated directly into anti-CD20, a small molecule drug (CG1), or secreted insulin and transferrin. In all four cases, LUX-MS demonstrated high selectivity. In 2022, the Merck team reported the system named PhoTag where a small molecule photocatalyst flavin was conjugated to antibodies and used to produce phenoxyl radicals.^159^ By using phenol-biotin with msec half-life,^205^ they showcased the system for long-range labeling using a tandem secondary antibody strategy and achieved on-cell biotinylation on PBMC cells and at PD-1/PD-L1 cell synapses. However, both the photosensitized singlet oxygen species and the phenoxy radicals have relatively long half-lives of 3.5 μs^158^ and the latter at 1 ms,^164^ respectively, which have lower resolution than more reactive photoprobes such as aryl-diazirines and aryl-azides.

More recently, we established a multiscale photocatalytic PLP platform, called MultiMap,^19^ using a single photocatalytic dye, Eosin Y (EY). When EY is illuminated with blue or green light, it is capable of triggering photoactivation of all the commonly used photoprobes (Figure 4B). This xanthene-scaffold photocatalyst has been widely used in polymer science and for photoredox synthesis given its low cost and commercial availability, currently ∼ 100-fold cheaper compared to Ir catalyst.^235,237^ We discovered that EY can efficiently activate aryl-diazirine-biotin labeling upon blue light illumination, and also triggers labeling with three other photoprobes such as aryl-azide-biotin, phenol-biotin and singlet oxygen/hydrazide. This allows us to use one single photocatalyst to activate multiple photoprobes with different labeling radii in one workflow. A facile two-step bioconjugation method was developed to directly incorporate EY onto primary antibodies with tunable resolution depending on the labeling radii of the photoprobe used. We applied MultiMap PLP to probe the off-state of the EGFR neighborhood by conjugating EY onto the therapeutic antibody cetuximab that competes with EGF.^238^ We identified 29 high-confidence neighbors including a phosphatase PTPRF capable of dephosphorylating the EGFR, as well as some tumor promoters and tumor suppressors. We also labeled some proteins known to associate with the inner leaflet of the membrane. This was not surprising given the neutral charge of the photoprobes used and their diffusive distance once triggered. More than half of the 29 candidates were validated using two orthogonal methods such as immunoprecipitation and AlphaFold-Multimer binary protein model prediction.^239^ The MultiMap platform, coupled with routine validation, can greatly streamline future studies on protein neighborhoods and provide additional validation or structural insights through complex prediction.

MultiMap was also shown useful to identify cell–cell interactions at immune synapses. Robust labeling was found for the longer phenol-biotin probes but not the shorter-range aryl-diazirine or aryl-azine probes highlighting the need for long and short-range probes. We tested the neighborhoods for immune synapses both induced by bispecific T cell engagers (TCE) and chimeric antigen receptor T (CAR-T) cells. The T cell neighborhoods were remarkably similar; the strongest hits in our PLP experiments identified the well-known intracellular kinases, ZAP70 and LCK, as well as phosphatase PTPN6. MultiMap allows us to monitor the cell surface and cell–cell neighborhoods on the molecular level analogous to Google Maps at the macro-scale. We further expanded this platform to a new set of workflows, extracellular MultiMap (eMultiMap) and intracellular MultiMap (iMultiMap), in a new study to visualize dynamic EGFR neighborhoods.^240^ By incorporating EY selectively to EGFR using extracellular Flag-Tag and intracellular HaloTag, which are orthogonal to EGFR signaling, we tracked neighborhood changes upon EGF ligand stimulation and identified >300 EGF-dependent neighbors of EGFR during different stages of signaling. This work greatly expanded the targetable proteome that is independent of antibody selectivity and intracellular permeability, which provided an alternative method to study dynamic interactomes in live cells.

Many new organic photocatalysts have been discovered or deployed for photocatalytic proximity labeling, including JF570 demonstrated by the Backus lab for both surfaceome and intracellular targeted labeling.^241^ The Moellering group, for example, demonstrated in 2024 an approach to study integrin-family interactions using lumichrome-conjugated RGD ligands.^242^ By triggering phenol-biotin labeling, they compared the interactome in different cell types and cellular microenvironments, and eventually demonstrated in pre- and post-metastasis triple negative breast cancer cells. Scaffolds such as deazaflavin was also showcased by the Hackenberger group to trigger the activation of diazirine, aryl-azide and phenol.^243^ During the preparation of this review, several new photocatalytic PLP have emerged in the literature, highlighting both the urgent need for and rapidly evolving nature of interactomics methods in this field.^495−499^

### 3.2 Outlook

#### 3.2.1 Remaining Challenges for Interactomics Studies

The development of PLP methods for defining protein neighborhoods on the cell surface is in its infancy. While these studies show what is proximal to the POI, we have not fully addressed functional interactions. Functional genomic screens will be very useful for defining the importance of new and established proteins in surface neighborhoods. Proteome-wide interactions can also be screened using genetically engineered libraries.^244,245^

How best to validate neighborhood results? It is critical to have good controls for PLP experiments given the highly packed nature of the surfaceome. These ideally will have a matched nonspecific surface labeling control, or plus/minus ligand binding to discern what neighbors are induced by ligand binding. It is also necessary to use orthogonal methods to validate physical interactions. This often involves bespoke *in vitro* reconstitution, structural analysis, or binding assays. However, given the number of hits from a proteomics experiment, it can be difficult to perform this validation workflow in high throughput. In the recent MultiMap study,^19^ we used routine (but expensive) co-immunoprecipitation as biochemical validation and coupled with recently introduced AlphaFold-Multimer as two orthogonal validation routes. The latter is potentially scalable and will certainly grow in rigor and speed, but we need a more scalable biochemical/biophysical assay for direct binding.

With ever-enhanced profiling resolution, it has become possible to visualize interactions in cells with higher spatial resolution using various PLP methods. With recent and existing studies focusing on intracellular subcellular organelles such as the ER^241^ or stimuli-triggered cellular aggregates such as stress granules,^232^ specific regions of the cell surface could also be targetable, including the membrane microdomains. Often known as lipid rafts,^246^ these cell surface regions are typically enriched in cholesterol and sphingolipids. Understanding the protein neighborhoods in this specific region could be helpful to elucidate the complete mechanism behind signaling transduction, cell–cell recognition, or protein sorting.^247−249^

So far, most interactomics studies are using *in vitro* cell culture systems with large numbers of cells (typically 5–10 million cells per sample). Cell lines are useful but do not represent the heterogeneity or architecture of diseased tissues, tumors, or healthy cells. A next step for more biological relevance would be to conduct these studies in more complicated samples such as organoids, xenografts, or even human tissue samples during various biological processes.^203,233,234,250−501^ At the same time, single-cell proteomics is beginning to show some advantages over traditional bulk analysis methods.^251^ However, given the surfaceome is 100-fold less abundant than the cytosolic proteome, it may still be a while before we can get adequate surfaceomic coverage at the single-cell level. However, one can identify candidates in bulk and then probe by more sensitive immunological means on those specific targets. Though RNA levels may not be directly correlated to protein expression, single-cell RNA-seq can also facilitate the discovery process since it can provide heterogeneous RNA-level signatures and features. It is becoming more routine to study intercellular interactions in heterogeneous biological systems using single-cell transcriptomics.^252,253^ We believe future advances in mass spectrometry and single-cell proteomics may enable closer examination of protein neighborhoods in patient samples.^254^

#### 3.2.2 Opportunities for Future Studies

Proteomics studies produce large amounts of data sets and information. Thus, generating publicly accessible community resources is equally important for a better understanding of protein interactions. Examples such as the humancellmap.org(168) or the OpenCell system^255,256^ present searchable and categorized information of protein localization on a proteome-wide level. For protein interactions, researchers are routinely using STRING^257^ or BioGrid^258^ to find evidence from previous studies. An integrated platform that contains curated PLP information of direct physical interactions of proteins will significantly help researchers to better understand surface interactomes.

Artificial intelligence has made a huge impact in the structural biology field with AlphaFold2 and other iterations including AlphaFold-Multimer for predicting binary complexes,^259^ ColabFold,^260^ RoseTTAFold-All-Atom,^261^ and introducing model parameters such as pDockQ,^262^ pDockQ2,^263^ and SPOC.^264^ Newly demonstrated AlphaFold3 can potentially bring many new opportunities in the field with its unprecedented accuracy.,^265^ along with other tools in the field.^266,267^ We believe proteome-wide screening data sets such as PrePPI^268^ or Predictome^264^ can greatly assist future work studying cell surface interactome and provide structural insights for therapeutic development.

## 4 Extracellular Post-translational Modifications in Health and Disease

In addition to changes in expression levels and new interactomes, the surfaceome is intricately regulated by post-translational modifications (PTMs). Proteoform diversity across the human proteome increases exponentially with PTMs, which represent at least 600 different reactions, and greater than 100,000 modified amino acid sites.^269^ The vast majority of PTMs are intracellular and reversible by the enzymatic action of writers and corresponding erasers. In contrast, there are only about a score of extracellular PTMs and most are irreversible due to the lack of erasers. Extracellular PTMs are dominated by glycosylation, proteolysis, and disulfide formation, in addition to less abundant phosphorylation, citrullination, lipidation, and sulfonation among others. Thematically, extracellular PTMs can contribute to ligand–receptor interactions, membrane protein complexes, small molecule recognition, protein stability, and protein function. Upon dysregulation, extracellular PTMs can contribute hallmark pathological characteristics in cancer, immune disorders, inflammatory conditions, and microbial infections which can be useful biomarkers.

The state-of-the-art proteomic technologies are beginning to interrogate key surfaceome PTMs under various cellular environments. This is an expanding field where new technologies are beginning to confront the analytical challenges of heterogeneity, low abundance, partial stoichiometry, and sometimes poor biophysical properties of cell surface proteins that can preclude in-depth analyses. Here we highlight important chemical biology and engineered protein tools to identify extracellular PTMs. We then discuss new therapeutic opportunities to target disease-associated PTM neo-epitopes and the cell surface enzymes that modify the surfaceome.

### 4.1 Cell Surface Glycosylation

**Figure 5:**
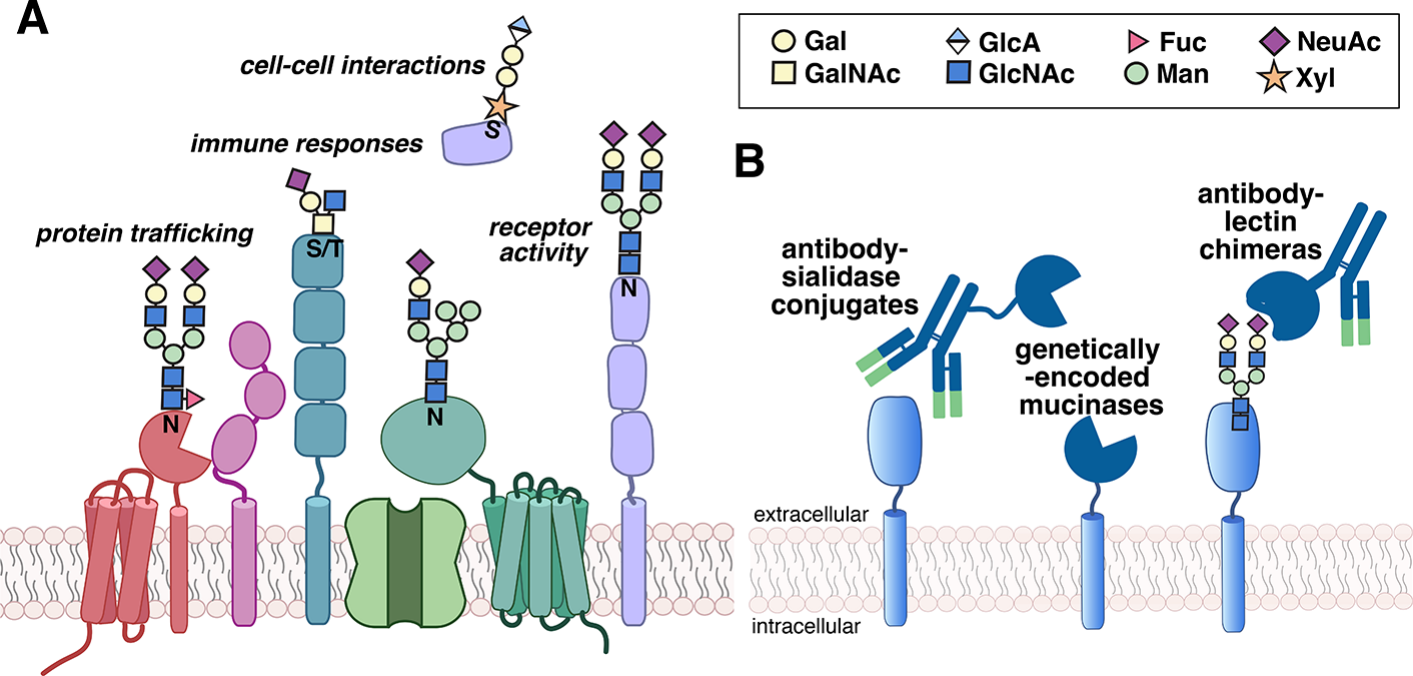
Protein glycosylation, the major PTM on the cell surface introduces heterogeneous oligosaccharides onto the cell surface. (A) Glycoproteins account for 80–90% of surfaceome. Complex oligosaccharides are attached to asparagine (N) or serine/threonine (S/T) residues on the peptide backbone. (B) Emerging therapeutic modalities based on engineered proteins and antibodies which target and manipulate the cell surface glycome.

Encasing nearly all living cells, glycosylation represents the most abundant PTM displayed on the cell surface. Protein glycosylation is a highly complex and heterogeneous PTM that decorates 80–90% of membrane-embedded and extracellular proteins (Figure 5A).^77,270^ With hundreds of glycosylation biosynthetic enzymes, there is an estimated ∼ 10^12^ possible branched carbohydrate structures that can exist among glycoconjugates.^271^ Glycans are organized into subtypes based on the type of amino acid residues modified, and the chemical features within glycan structures. The vast majority of extracellular protein glycosylation falls into two categories: asparagine-linked (N-glycosylation) and serine/threonine-linked (*O*-linked) glycosylation. Other cell surface glycoproteins feature glycosylphosphatidylinositol (GPI) modifications.^272^*N*-glycans fall into three major types that include oligomannose-, complex-, and hybrid-type glycans linked to a common pentasaccharide core structure and a peptide-linked N-acetyglucosamine (GlcNAc).^273^ Conversely, *O*-linked glycans are attached to proteins through a variety of sugars including N-acetyl-d-galactosamine (GalNAc), GlcNAc, L-fucose (Fuc), d-glucose (Glc), D-mannose (Man), or D-xylose (Xyl) moieties (see key, Figure 5).^43,274,275^ Extracellular, mucin-type *O*-glycosylation is the most abundant subtype, and features several variable core structures bound to a GalNac linked sugar.^276^ Most surface proteins contain an average of three glycosylation sites, but there are protein families that are densely glycosylated. Heavily *O*-glycosylated proteins, for instance, are called mucins, and they create a mucosal barrier around epithelial cells.^275,277^ Proteoglycans, defined as glycoproteins that are heavily modified with O-linked glycosaminoglycans (GAGs), are another major group of extracellular glycoproteins that are also present in the extracellular matrix.^278^

The vast majority of extracellular glycans are assembled, modified, and attached to proteins during the secretory pathway. This process comprises the coordinated activities of approximately 200 glycosylation enzymes and 173 of these are known to create complex glycan structures.^43^ Depending on environmental and cellular cues, these enzymes introduce heterogeneity based on how often protein sequences are glycosylated and the different carbohydrate linkages at a single glycosite. While the vast majority of glycosylation enzymes are active intracellularly, there are also disease- and cell- type associated extracellular transferases and hydrolases that are capable of further tailoring cell surface glycans. Activated platelets, for example, release extracellular, active glycosyltransferases. In another example, cancer cells can induce apoptosis by overexpressing and releasing sialidases that deglycosylate and activate the Fas ligand.

Glycoproteins constitute a major component of the dense, nanoscale external cell structure named the glycocalyx.^274^ The surfaceome status in health and pathophysiology are profoundly affected by alterations within the structures of glycans and glycosylation density not only within the entire glycocalyx structure but also individual glycoproteins. Proper protein glycosylation is an important characteristic for protein solubility, stability, protein–protein interactions, and protein functions within the surfaceome (Figure 5A). Cell surface glycans can modulate cell signaling, intercellular interactions, immune activities, and the cellular response to its surrounding environment. Changes in protein glycosylation during synthesis is a hallmark of many pathophysiological conditions that includes cancer,^279,280^ inflammation,^281,282^ infection,^283^ and neurological disorders.^279,284^ Cancer cells, for example, will hyper-sialylate their cell surfaces.^10,285,286^ Sialic acid, a 9-carbon monosaccharide present on the termini of most cell surface glycans, interacts with Sialic-acid binding immunoglobulin receptors (Siglecs) that can enable cancer cells to evade immune checkpoints.^287^ In the EGFR/HER2 tyrosine kinase family, tumor cells will reprogram glycosyltransferases that remodel the typical glycosylation patterns of these receptors, which can promote aberrant signaling.^288−290^

#### 4.1.1 Proteomic Approaches to Study Glycosylation on the Surfaceome

Glycoproteomics has emerged as a powerful analytical technique to unravel how glycoproteins diversify the surfaceome in health and disease. For detailed perspectives of glycan and glycoprotein characterization using different glycoproteomic techniques, we recommend these comprehensive reviews.^291−295^ Here, we will briefly discuss the advantages of using chemical, chemical-biology, and protein-based approaches that have enabled the enrichment of glycoproteins for proteomic identification.

As mentioned above, to enrich and identify cell surface glycoproteins, chemical enrichment strategies were first developed, such as hydrazide-coupled resins to capture aldehydes generated by mild oxidation of cis-diols present within carbohydrate chains such as sialic acids, a terminal sugar moiety on most extracellular glycosites.^76^ Other chemical-based approaches include hydrophilic interaction liquid chromatography (HILIC) that takes advantage of the hydrophilic nature of glycans,^296,297^ and electrostatic repulsion-hydrophilic interaction chromatography (ERLIC) that exploits the charged states of sugars.^298^ Alternatively, glycosyl enzymes can be leveraged to introduce chemical labels onto extracellular glycans. Paulson and co-workers showed that galactose oxidase (GAO) generates aldehyde-containing galactose sugars which can be further biotinylated using oxime chemistry.^299^ In combination with GalT1, which transfers galactose to terminal GlcNAc residues, this approach has been widely used to enrich O-GlcNAc residues.^270,300^ Sialyltransferases (e.g., St6Gal1 and St3Gal1) have also been used to label glycans with chemical handles that include azido- or biotinylated-motifs.^301−304^

Another conventional glycoprotein enrichment strategy entails the use of carbohydrate-binding proteins (lectins) and antibody resins to pull-down glycoproteins and specific subtypes of glycoproteins. These are typically weak interactions. By using high avidity commercial formats, however, lectins such as concanavalin A (ConA), wheat germ agglutinin (WGA), and Jacalin A (JAC) have been widely used to enrich and identify cohorts of N- and O-linked glycopeptides.^293^ Due to their low affinity and promiscuous interactions among glycan motifs, lectins are useful at broadly identifying different carbohydrate motifs, but their poorly defined specificities may be a disadvantage of characterizing specific glycosylation subtypes. In recent years, Bertozzi and co-workers conjugated an inactive O-glycoprotease (StcE) to resin, which allowed them to pulldown and identify mucin-domain-containing glycoproteins.^305,306^ Galectins and Siglecs, in other examples, have been used in a similar manner to identify specific glycosylated ligands.^307,308^

Lastly, there are several chemical biology approaches that exploit the endogenous biosynthetic glycosylation machinery to introduce specific unnatural sugars with chemical handles. In metabolic oligosaccharide engineering (MOE) strategies, sugar precursor building blocks are replaced with clickable sugar analogs using the endogenous glycosylation machinery.^309,310^ Alkyne- or azide-modified ManNAc analogs, for example, endow terminal sialic acids with clickable chemical handles for subsequent enrichment and pull-down experiments. MOE approaches using a range of unnatural sugar precursors (ManNAc, GalNAc, GlcNAc, fucose) have been developed. An important caveat to this approach, however, is that metabolic crosstalk and enzyme tolerance of unnatural functionalization can limit the specificity and degree of chemical labeling over protein glycoforms. Incorporation of azido-fucose, for instance, can inhibit or limit protein fucosylation. Due to metabolic crosstalk, GalNAc analogs are incorporated generally into O- and N-linked glycans. Engineered GalNAc transferases alongside a paired GalNAc donor, however, will selectively label O-glycosylated proteins.

With an improved understanding of surfaceome glycosylation and its role in human health, there are many promising potential therapeutic avenues (Figure 5B). Taking advantage of the aberrant hyper-glycosylation as a hallmark of cancer, an area of therapeutic exploration has centered around glycoengineering strategies to manipulate cancer-regulated neo-glyco-epitopes. By conjugating a bacterial sialidase to trastuzumab (a HER2- targeting antibody), Bertozzi and co-workers were able to remove sialoglycans from the cancer cell surfaces, thereby removing the Siglec interactions and inducing an antitumor response.^127,311^ Similarly, Paszek and co-workers displayed the mucinase StcE to NK cells by genetically encoding a leucine zipper, and then showed that StcE-displayed NK cells induced cytotoxicity toward cancer cells expressing high levels of mucins.^312^ Wu and co-workers designed sialidase-fusions onto bispecific T-cell engages (TCE) to remove sialic acids from the T-cell tumor interface, which enhanced T-cell cytolysis.^313^ More recently, Bertozzi and co-workers engineered antibody-lectin chimeras that contain a tumor-targeting arm and a lectin decoy arm, which prevents tumor cells from escaping immune checkpoints.^314^

### 4.2 Extracellular Proteolytic Remodeling

Another widespread cell surface modification is site-specific extracellular proteolysis, an irreversible molecular switch generated by peptide bond hydrolysis. Unlike the mostly static state of extracellular glycosylation, extracellular protein function can be modified either by regulated proteolysis at the cell surface or in transit through the secretory process. There are approximately 600 proteases encoded in the human genome and most comprise membrane-bound or soluble proteases.^315^ At minimum, there are 70 well-annotated extracellular active proteases in addition to noncanonical proteases that can relocate to the cell surface.^316^

**Figure 6:**
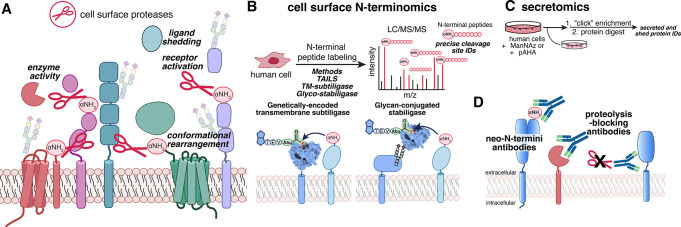
Cell surface proteolysis is an irreversible modification catalyzed by dozens of secreted- and membrane- anchored proteases. (A) At the cell surface, proteases (scissors) specifically cleave within extracellular domains resulting in many functional consequences across the surfaceome. (B) Cell surface N-terminomics has emerged as a powerful tool to identify neo-N-termini that remain after proteolytic cleavage. To globally identify cleavage sites across living human cells, our group engineered cell surface subtiligases, an engineered peptide ligase. (C) Complementary to cell surface N-terminomics, secretomics is another popular proteomic approach for identifying the soluble products of cell surface proteases. To identify secreted proteins, a number of metabolic and proximity-labeling approaches have been developed. (D) Having identified neo-proteolyzed surface targets it is has been possible to engineer antibodies that block or selectively target these surface proteins as a potential therapeutic.

Extracellular proteases usually catalyze site-specific proteolysis of the target as opposed to complete degradation of proteins that occurs in the lysosome or proteasome. Extracellular proteases usually cleave cell surface proteins at controlled positions, that can trigger changes in protein localization, protein–protein interactions, and protein activities (Figure 6A).^317^ The functional consequences for most of these proteolytic events have not been studied in detail. However, for those rare examples that have been studied, proteolysis results in a gain-of-function phenotype.

Proteases play essential roles in biological signaling and upon dysregulation, play pivotal roles in pathophysiological outcomes.^318,319^ Aberrant proteolytic remodeling is a hallmark of inflammatory diseases which includes CNS disorders and cancer.^317,320,321^ In contrast to most PTM-enzymes, dozens of proteases actively tailor the surfaceome on or between cells at the plasma membrane. Proteolysis at the plasma membrane is predominantly responsible for the maturation, trafficking, shedding and contributes to endocytosis of surface proteins.^316,322,323^ On the cell surface, regulated proteases will tailor specific membrane proteins. Within the endoplasmic reticulum and golgi-apparatus, signal peptidases process signal peptides and pro-protein convertases can activate proteases by cleavage of their pro-domains on zymogen precursors (for review see references^324, 325^)

Extracellular proteolytic tailoring, both on the cell surface and in the extracellular space, is mediated through a highly regulated, complex signaling network surrounding a cohort of secreted and membrane-anchored proteases. The extracellular protease network, comprises members of all major catalytic protease types: cysteine, metallo-, aspartic, threonine, serine proteases which are expressed in a cell-type and tissue-dependent manner alongside protease inhibitors and protein interactors.^322^ Among their many and variable biological roles include remodeling the architecture of the cell surfaceome and the extracellular matrix (ECM). Serine- and metalloproteases are the two major protease families, comprised of mainly endopeptidases that discretely cleave within extracellular regions and can shed extracellular protein domains. Classical sheddases, like the ADAMs protease family, cleave extracellular proteins within flexible, membrane-proximal regions, releasing large soluble protein domains from the membrane.^323,326^

Paracrine and autocrine signaling cascades are triggered by proteolytic shedding. Proteases are produced as inactive zymogens that are activated by proteolysis either in cis or trans. They then cleave and often shed membrane-bound proteins to create biological active signals that include cytokines (e.g., IL-1 family)^327^ and growth factor receptors (e.g., hGH, IGF-1).^328^ Transmembrane, single-pass receptors, and multipass membrane proteins are also functionally regulated by extracellular proteases, which tune their ligand-binding properties and complexation via proteolytic shedding. For example, extracellular proteases cleave and activate receptors that include single-pass receptors such as Notch and multipass receptors such as the protease-activated receptor (PAR).^329,330^ Extracellular proteolysis is also a global modification that impacts the signaling activities of functional proteins such as transmembrane kinases and phosphatases, cytokine receptors, and phagocytic receptors are broadly regulated through proteolytic shedding events.^331−335^ Among these transmembrane receptors, extracellular proteolysis may also trigger a proteolytic cascade driven by intra- membrane and cellular proteolysis, which reorganizes the locations of protein fragments for downstream cell signaling. For example, ADisintegrin And Metalloprotease (ADAM) are sheddases that cleave membrane proximal regions, which then trigger intramembrane cleavage by gamma-secretase and subsequent intracellular proteolysis.^326^ Notch signaling, for example, upon mechanical or ligand activation induces membrane-proximal cleavage followed by intracellular proteolytic processing during signal transduction.^334^ Intriguingly, proteolytic shedding plays a dual, contrasting role across pathologies. In cancer, sheddases including ADAM metalloproteases and matrix metalloproteases are highly dysregulated, either down- or up- regulated.^317,318^

#### 4.2.1 Proteomic Approaches to Study Proteolysis on the Surfaceome

Several approaches have been developed to characterize proteolysis outside of the cell. A powerful approach for elucidating protease substrates has been N-terminomics, a proteomic technology that tags and identifies neo-N-termini generated at precise cleavage sites.^336^ To introduce chemical tags onto N-termini for proteomic identification, several chemical (e.g., 2PCA,^337^ TAILS,^338,339^ COFRADIC^340^) and enzymatic (subtiligase^341−345^) approaches have been developed. At present, chemical-based approaches have enabled coverage of intracellular proteolysis but have rarely identified extracellular protease substrates. In a few examples, TAILS (Terminal Amine Isotopic Labeling of Substrates), a strategy developed by Overall and co-workers which involves fractionating N-terminal peptides from internal peptides by differential chemical modification before and after proteolysis. For instance, TAILS N-terminomics was employed to identify metalloprotease substrates within fibroblast secretome samples, as well as the cell surface proteins cleaved by secreted cathepsins in pancreatic tumor samples.^339,346^ Importantly, isolating extracellular fractions and isolation of membrane samples was necessary for TAILS-based enrichment for neo-N-termini on extracellular proteins. Nevertheless, global characterization of surfaceome proteolysis using these approaches has remained difficult due to the fact the surface proteome is far less abundant than cytosol as mentioned above.

Another widely employed N-terminomics strategy is dependent on an engineered N-terminal ligase (subtiligase) that will catalyzes transfer of peptide ester specifically onto the α-amine and not ε-amine of another peptide or protein.^347^ By mixing a biotinylated and mass-tagged peptide ester substrate in the presence of protein mixtures, subtiligase will transfer the tagged peptides specifically onto N-terminal amines of proteins for enrichment and proteomic analysis.^347^ Like other N-terminomics methods, subtiligase-based strategies are widely successful at globally capturing soluble proteolytic modifications either inside or outside the cell, such as for caspases or serum proteins.^342−344,348,349^ Soluble subtiligase-based N-terminomics was not well-suited for characterizing cell surface proteolysis due to inefficient labeling.^350^ However, by localizing the subtiligase to the extracellular plasma membrane environment, we increased the extent of N-terminal labeling by 50-fold allowing efficient enrichment of surface proteolyzed proteins (Figure 4B).^14^ In our first cell surface N-terminomics strategy, we generated a genetically encoded subtiligase that when displayed on HEK293T cells, broadly labeled hundreds of neo-N-termini.

Most recently, we developed a second-generation of cell surface N-terminomics that does not require cellular engineering to attach subtiligase to the cell surface.^351^ This approach took advantage of the glycan density on cell surfaces to covalently tether an aminooxy-conjugated subtiligase broadly across cell surface glycans. Glycan-tethered subtiligase allowed us to sample and identify neo-N-termini across human cell types including cancer cell lines and primary immune cells. To date, cell surface N-terminomics using tethered subtiligase has revealed more than 1,500 cell surface neo-N-termini across 500 different surface proteins for which >90% were previously undocumented even from large N-terminomics databases.^352^

Using cell surface N-terminomics on isogenic nontumorigenic cell lines transformed with single oncogenes, we observed bidirectional changes where some proteolytic events were up-regulated by the oncogene and some were down-regulated.^14^ This occurs by the oncogene inducing changes in the expressed and activated proteases. Indeed, the cell surface N-terminomics also revealed propeptide processing within extracellular proteases, giving clues to changes in the abundance and activities of these enzymes.

#### 4.2.2 Proteomic Approaches to Study Secreted Proteins in the Secretome

Complementary to cell surface N-terminomics, secretomics is another widely used proteomic approach that can identify proteolytically shed and secreted proteins within the extracellular milieu. To overcome the abundant contamination from serum-derived proteins, secretomics employs specialized experimental workflows to specifically enrich for secreted or shed proteins. There are two widely used metabolic strategies for identifying secreted proteins within serum-containing media. In one approach pioneered by Lichtenthaler and co-workers, azido-functionalized sugar are metabolically incorporated into the secreted glycoproteins for a click-chemistry based enrichment.^353,354^ To identify additional nonglycosylated secreted proteins, another strategy developed by Krijgsveld and co-workers entails the addition of pulsed azido-homoalanine for protein enrichment. To identify secreted proteins *in vivo*, Long and co-workers have encoded secretion tags onto proximity labeling enzymes, which then allows proteins to be labeled and enriched from blood plasma.^355^ Unlike cell surface N-terminomics, an important caveat to secretomics is that it does not provide positional information on proteolytic modifications without N-terminal enrichment, and may miss small shed domains if peptide coverage is sparse.^323^ Recently, we have combined subtiligase-based N-terminomics with secretomics to characterize how hypoxia affects proteolytic activities and shedding relative to normoxia in pancreatic cancer cell lines.^356^

#### 4.2.3 Targeting Cell Surface Proteolytic Events with Engineered Antibodies

Given the fundamental roles of proteases in surfaceome biology, there has been tremendous attention to leveraging extracellular proteolysis for therapeutic strategies using engineered antibodies. Protease-targeted therapeutics are increasingly explored as anticancer agents due to the critical roles of dysregulated, overly active proteases within the tumor microenvironment (TME). In the past few decades, numerous groups have explored using small molecule inhibitors, peptide substrate inhibitors, and antibody-based activity blockers to inhibit proteases that contribute to carcinogenesis and therapeutic resistance.^317^ Proteases can be complicated pharmacological targets due to their semiredundant roles in a multitude of biological signaling pathways. Most recently, antibody-based protease blockers have garnered attention because they offer potentially higher selectively toward specific proteases and may have more success in circumventing off-target effects.^357^

Taking advantage of the high activity of disease-regulated proteases, others are developing protease-activatable therapeutic strategies. Specifically, these modalities serve to deliver biologics and prodrug modalities more specifically to cancer cells that exist in TME with enriched cleavage activities. Protease-activated antibodies, called pro-antibodies, that are only unmasked for binding to the target in the TME can selectively target tumors more successfully relative to traditional antibodies.^358,359^ Following a similar logic, caged peptide therapeutics have shown success in tumor penetration and drug delivery via proteolytic processing.^360^ These approaches offer precision-based medicine therapeutics depending on the proteolytic expression patterns among patients and cancer types.

Disease-regulated extracellular proteolysis can also provide unique proteolytic neo-epitopes for immunotherapeutic avenues (Figure 6C). One approach is to selectively block the aberrant shedding of cell surface proteins. Wucherpfennig and co-workers, for example, selected antibodies to selectively block the proteolytic shedding of MICA/B, a T-cell and NK cell ligand that is highly shed from many cancer cells so that they can avoid immune recognition.^361^ Likewise, vaccine-generated antibodies can also block the MICA/B shedding, and may provide a promising approach for cancer vaccines. Our group has also recently developed a phage display-based epitope-directed strategy (EDS) to develop antibodies that block proteolysis.^362^ We showed the versatility of this approach by developing proteolysis blocking antibodies against two cancer-dysregulated cell surface receptors, CDCP1 and EphA2, and four different metalloproteases.^362^ Furthermore, we have also developed antibodies that selectively bind the proteolyzed, neo-epitopes that remain on cell surfaces. Lastly it we found a poor correlation between the oncogene-induced expression of surface proteins and the up or down-regulation of the proteolytic events associated with them. By targeting RAS-driven cancers that display high amounts of cleaved CDCP1 with antibody-drug conjugates, BiTES, and radionuclide-antibody conjugates, we have shown that these antibodies provide a greater therapeutic index.^138,363^ With emerging information on precise cell surface cleavage sites (neo-N-termini), biologics that target proteolytic neo-epitopes is an exciting therapeutic landscape.

Finally, there is a fundamental and complex interplay between proteolysis and glycosylation, which are the major PTMs on the cell surface. Most often, glycosylation sites will block cleavage events and there are many examples of highly glycosylated protein domains within the membrane proximal regions that prevent extracellular shedding. As an example, O-glycosylation isoforms of TNF-alpha coregulate its proteolytic shedding during activation.^364^ In other circumstances, glycosylation may not interfere with proteolysis, as recently demonstrated with the ability of the cathepsin family to cleave O-glycosylated peptide regions.^365^ With improved coverage of glycosylated and proteolytic modifications in the future, we can further appreciate the balance between these extracellular modifications.

### 4.3 Additional Cell Surface Post-translational Modifications

In addition to structurally complex modifications, transmembrane proteins and extracellular soluble proteins are also tailored with relatively small chemical motifs. Among these are extracellular PTMs such as disulfide bond oxidation, lipidation, phosphorylation, and citrullination.^366^ Most PTM-enzymes modify predestined surfaceome proteins during intracellular trafficking. Nevertheless, there is emerging evidence that PTM-enzymes may actively remodel proteins within the plasma membrane. For example, extracellular kinases and phosphatases can be secreted as soluble enzymes or within extracellular vesicles. The evidence for extracellular phosphorylation activities is an exciting and emerging area in cancer^367^ as extracellular ATP is highly increased (10,000–100,000-fold higher) in the TME.^368^ We have recently shown it possible to target a known ecto-kinase (casein kinase 2, CK2) to HER2 overexpressing cells by producing bispecific fusion, CK2-trastuzumab.^369^ This bispecific molecule overphosphorylates these cells, allowing the identification of new phosphoproteins on the cell surface. The resulting modified surfaceome also induced the production of antiphosphoprotein antibodies. In another example, citrullination, the loss of a positive charge on arginine residues, is catalyzed by intracellular and extracellular protein arginine deiminases (PAD). It was recently described that the secreted isoenzyme PAD4 catalyzes extracellular citrullination and plays an important role in inflammatory diseases including rheumatoid arthritis.^370^

Although our understanding of extracellular PTM dynamics is still nascent, emerging bioinformatic analyses and proteomic studies are expanding our knowledge of where and how these modifications contribute to surfaceome diversity. Whereas bioinformatic analysis can identify potential, PTM-accessible amino acids within extracellular domains, in silico approaches must be complemented with cell-based proteomic approaches to identify PTM differences in healthy and pathophysiological states. For example, Backus and co-workers recently developed a cysteine reactivity surfaceome profiling technology.^371^ At present, there are limited proteomic approaches tailored for globally characterizing extracellular PTMs outside of the cell but this field is rapidly changing.

## 5 Mapping the Immunopeptidome

The surfaceome is critical for the cell’s communication with the immune system. T cells constantly search for clues of “self” versus “non-self” and respond to threats such as cancer and infection.^372^ Key to this process is a set of peptides, collectively known as the immunopeptidome,^373^ that are displayed on the cell surface by the major histocompatibility complex (MHC). These molecules are divided into two classes, MHC-I and MHC-II, based on the subtypes of T cells they interact with.^374−376^ The nature of the peptidome directly impact the decisions made by T cells. Efforts to study the principles underlying peptide presentation have driven the development of biochemical and analytical tools for isolating and mapping the MHC immunopeptidome. Moreover, the potential to exploit peptide-MHC complexes (pMHC) therapeutically has spurred investigations of disease-associated immunopeptidomes. Below we will highlight the key developments and takeaways that shaped our understanding of the MHC-I peptide repertoire in health and disease. We also point the reader to excellent reviews discussing similar advancements for studying the MHC-II peptidome can be found in references^374, 377^.

### 5.1 The Basics of Immunopeptidome and MHC Presentation

MHC proteins are unique due to their extreme polymorphism in humans (>10,000 MHC alleles have been reported to date). Mammalian MHC-I proteins (also known as the human leukocyte antigen or HLA) are encoded at three genetic loci (*HLA-A, -B and -C*), and a given cell can possess 3–6 alleles at a time.^24,375^ The polymorphic residues are distributed across the peptide binding groove and dictate the types of peptides that can be displayed.^374,378^ Consequently, each allele displays a unique but often overlapping peptidome. Up to 10^6^ copies of MHC-I molecules can be found on most cells (the exact number depends on the cell line).^379−381^ It is estimated that 20,000 different peptides can be presented at levels varying from <10 to 1,000s of copies per cell.^24^ The large difference in number raises key questions on how peptide presentation is regulated, especially in the context of immune surveillance of self-versus-foreign peptides. Early biochemical and structural studies have revealed that MHC-peptide recognition is defined by a set of anchor residues (often at positions 2 and 9) flanking the displayed peptide sequence, typically 8–11 amino acids in length. This observation laid important groundwork for many analytical tools that are routinely used for analyzing MHC-I peptidome today.

**Figure 7:**
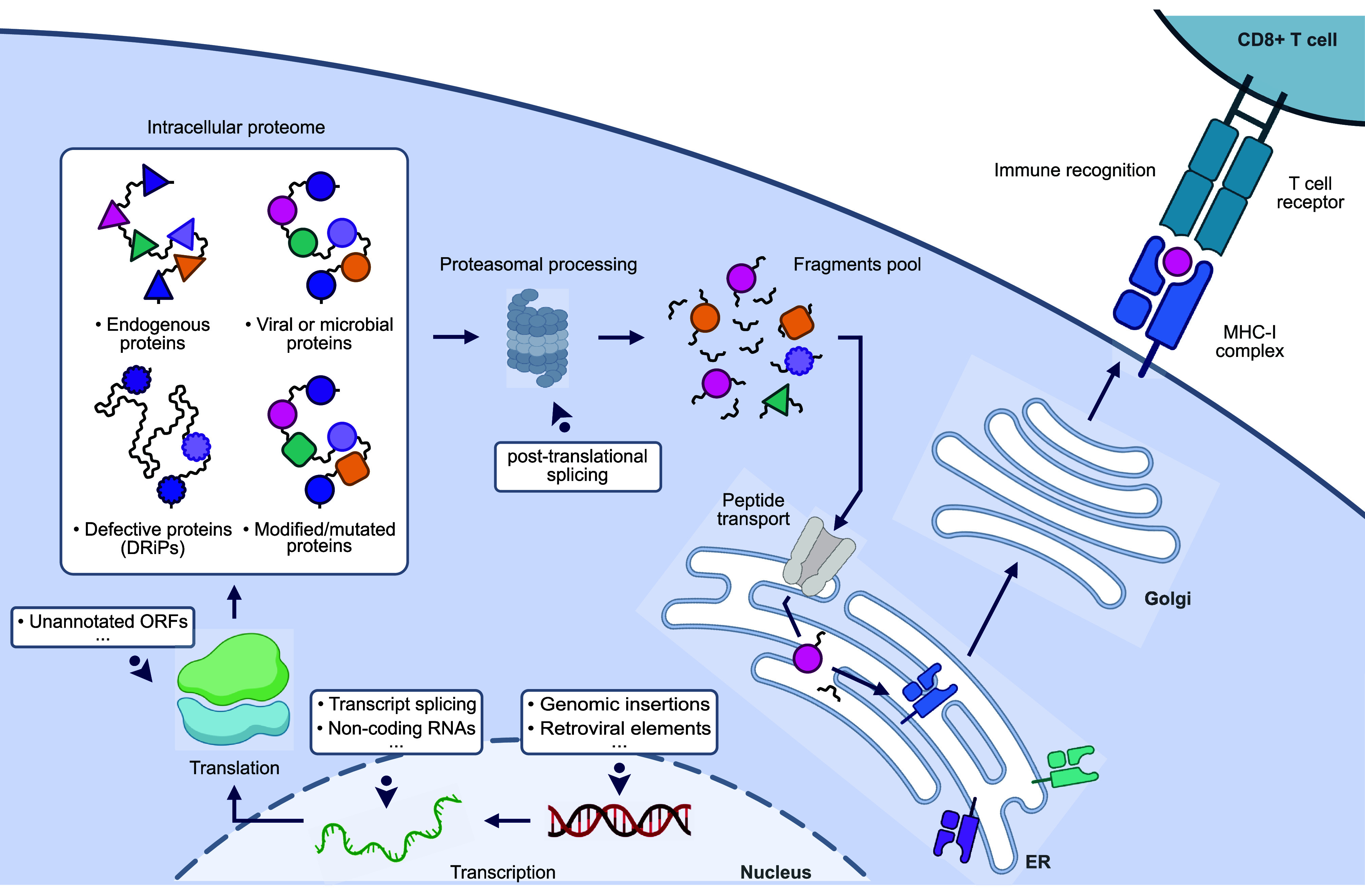
The basics of MHC-I peptide presentation. Intracellular proteins and polypeptides are degraded by the proteasome. Resulting peptide fragments are imported into the endoplasmic reticulum and loaded onto MHC molecules. The newly formed pMHCs undergo further editing and ultimately shuttled to the cell surface as ligands for T cell receptors on CD8^+^ T cells. A variety of proteins – self-or foreign, functional, or defected, modified or mutated – can contribute to the pool of MHC-I peptidome. Additionally, different disease associated genetic and transcriptional changes can produce unique peptides that are responsible for triggering an appropriate immune response. Portions of this figure were created with BioRender.com.

The majority of MHC-I peptides are generated from protein turnover mediated by the proteasome (Figure 7). Our understanding of what contributes to the immunopeptidome continues to evolve. The early view was pMHCs were generated when proteins reached the end of their lifetime.^372,382^ It is now recognized that a significant portion of the peptide repertoire is derived from defective ribosomal products.^372,383^ Other noncanonical cellular events can also contribute to the immunopeptidome.^375^ Re-expression of retroviral elements,^384^ genomic insertions,^372^ transcript splicing,^385^ and long noncoding RNAs^386^ have all been documented to produce antigenic peptides. Surprisingly, novel peptides are also generated post-translationally.^387^ Ligation between peptides (akin to protein splicing) can occur at the proteasome to give rise to antigens that are not genetically encoded. The percentage of spliced peptides in the immunopeptidome remains a subject of debate.^388,389^ Nonetheless, these alternative sources of MHC peptides underscore the complexity and need for deeper understanding of antigen presentation.

Direct sequencing of MHC-I peptides is a daunting yet necessary first step for studying and exploiting the immunopeptidome. Early efforts using mass spectrometry faced serval challenges. MHC molecules constitute only a small fraction of proteins in a cell (∼0.01%).^380,381^ In diseases such as cancer, they are further downregulated for immune evasion.^390^ Since most MHC peptides come from highly abundant proteins, disease-associated ones are extremely difficult to detect. The low copy number also imposes severe requirements on the sensitivity and specificity of pMHC recovery and analysis. Further complicating the issue, the diverse allotypes make pairing a peptide with the presenting allele challenging.^391^ Lastly, peptides are heavily edited at their N-termini by aminopeptidases before being transported onto a receiving MHC molecule.^24,375^ This processing step remains poorly understood and hinders the matching of a detected peptide sequence to a protein source. Despite these limitations, advances in mass spectrometry and computational technologies in the last 30 years have drastically changed the landscape of immunopeptidomics. Creative solutions have been developed to isolate and enrich for pMHCs from cultured cells and even patient samples. Many platforms are now available for researchers to dissect unique aspects of the immunopeptidome under specific biological contexts.

### 5.2 Methods to Isolate the Immunopeptidome

Studying the immunopeptidome has been enabled by innovations in technologies for extracting pMHC-I from the cell surface. These approaches can be thematically separated into two categories: mild acid elution (MAE) and affinity purification via antibodies. The former takes advantage of pMHC’s biophysical instability at low pH, while the latter requires a specialized set of reagents to capture complexes directly from the cellular milieu.^392^ Neither approach is perfect, and each has its own advantages and disadvantages. The following section highlights important lessons learned using different MHC recovery methods and areas for potential improvements.

#### 5.2.1 Mild-Acid Elution (MAE)

**Figure 8:**
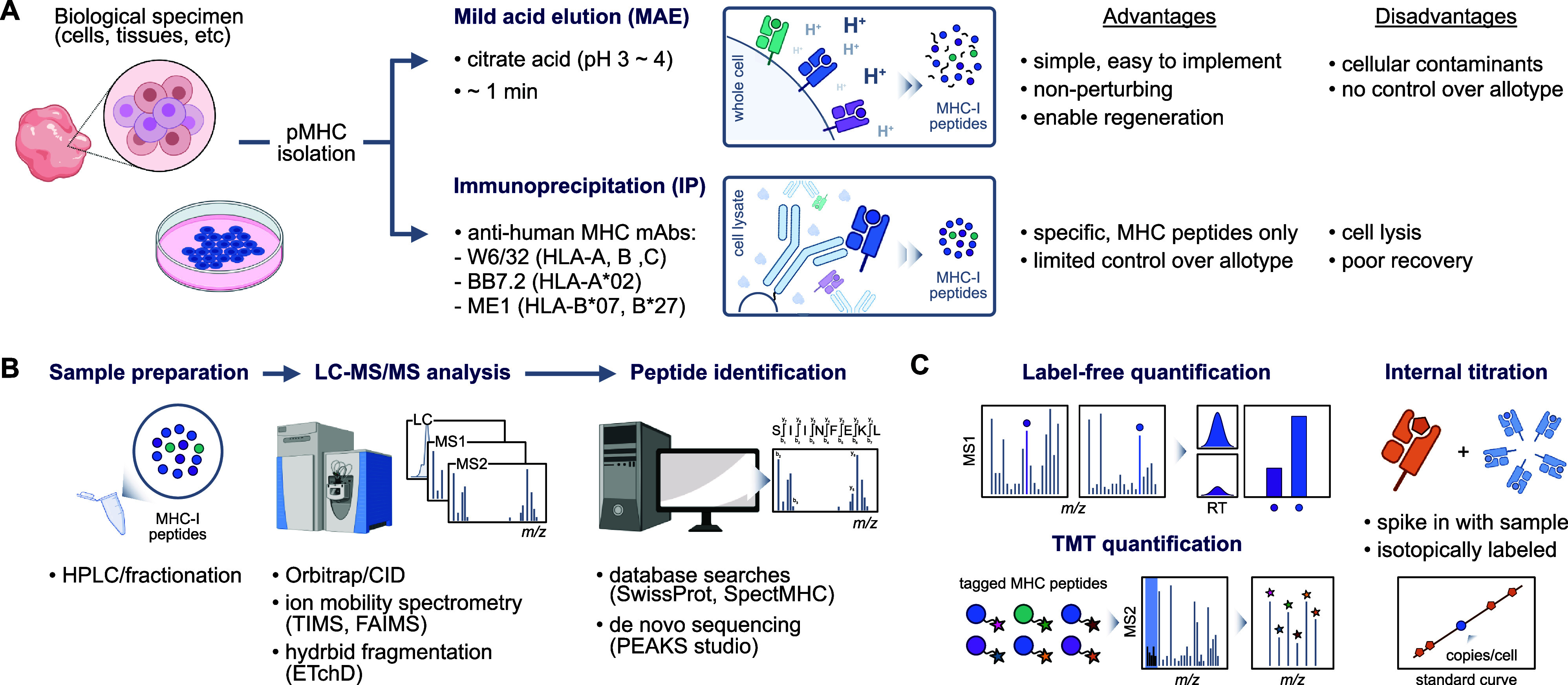
Profiling the immunopeptidome via mass spectrometry. (A) pMHCs can be isolated from primary tissues or cell lines using either mild acid elution or immunoprecipitation. (B) Workflow for MHC ligand identification. This process is similar to the ones used in conventional proteomics, but with a few adjustments. Different fragmentation methods and analysis software are used to account for the difference between MHC peptides and tryptic peptides obtained from proteolytic digestion. (C) Similarly, quantitative methods commonly used in proteomics can be applied to determine the relative and absolute copy-per-cell numbers for MHC peptides. Portions of this figure were created with BioRender.com.

Introduced by Sugawara and co-workers in 1987, MAE is one of the earliest and simplest methods to isolate the immunopeptidome (Figure 8A).^393^ Treating cells with acid (pH ∼ 3, typically with citrate buffer) causes the β2m to dissociate from folded pMHC, releasing the bound peptides in the process. This method was instrumental in early investigations of T cell responses to MHC peptides.^394^ MAE is rapid, and nontoxic.^395^ Consequently, it has been suggested as a method to monitor immunopeptidome regeneration and turnover. Thibault and co-workers used MAE to establish important features for neoplastic immunopeptidome: (1) most peptides are derived from highly abundant transcripts, and (2) a large portion (∼25%) of peptides found in neoplasia were absent in normal cells.^385^ Interestingly, unique peptides heavily reflect crucial molecules involved with pathways leading to neoplastic transformation. Despite the valuable insights gained, the acid eluate obtained from cells is often contaminated with non-MHC-I peptides, which reduces the number of identifiable pMHCs and complicates downstream analysis.^396^ For these reasons, in the last couple of decades, other recovery methods such as immunoprecipitation have been preferred for MS-based immunopeptidomics.

#### 5.2.2 Immunoprecipitation via Anti-MHC Antibodies

Immunoprecipitation (IP) using anti-MHC antibodies offers a more selective approach to enrich for MHC peptides (Figure 8A). Originally pioneered by Engelhard and Hunt,^397−399^ and later by Natheson^400^ and Rammensee,^401,402^ this method isolates the peptidome by first capturing pMHCs from cell lysates followed by acid dissociation. A handful of antibodies have been isolated for pMHC IP; however, the pan-specific clone W6/32 (first introduced by Hunt and co-workers^396^) remains the reagent of choice. One key feature of W6/32 is its capability to isolate all three classes of human MHC molecules (HLA-A, -B, -C). A disadvantage of a pan-specific pull-down is it captures of all pMHC molecules without retaining any allelic information. Researchers often rely on algorithms to assign peptides to specific alleles, which can introduce ambiguity and variability. In addition to W6/32, alternative antibodies with varying degrees of allele-specificity (e.g., BB7.2) have been used for isolating pMHCs. However, the allele coverage is neither complete nor sufficient. Many IP protocols also suffer from poor yield with losses up to 90% during recovery step and place demands on excessive sample size, in some cases up to 10^8^–10^9^ cells.^403,404^ While improvements have been introduced, most reagents and protocols are nearly identical to those used in Hunt’s seminal work almost three decades ago. As the demand for immunopeptidome analysis grows, better immunoprecipitation reagents and protocols are necessary to improve yield, bias, and robustness.

### 5.3 Instrumentation for Immunopeptidomics

The introduction of Orbitrap-MS marked a paradigm shift in the field and enabled facile identification of thousands of peptides in a single experiment.^405,406^ The sensitivity of modern MS instruments (e.g., Q-Exactive-HF, Fusion Lumos, Orbitrap Astral, etc)^396,407^ continues to lower experimental requirements for immunopeptidomics. Thousands of endogenous peptides can be detected in a single run using commercial mass spectrometers and standard workflows (Figure 8B). The improved sensitivity also enabled detection of rarer peptides carrying mutations and even PTMs.^408−410^ Despite improvement in sensitivity, standard immunopeptidomics protocols still require a large amount of cellular input (often up to 10^9^ cells) for reliable and compressive peptide identification.^391,396^ Recent development in advanced ion separation techniques (e.g., TIMS)^411,412^ and hybrid fragmentation (EThcD) methods^413,414^ are continually lowering the demand for input material. Carr and colleagues have reported an optimized method than enabled robust peptide detection with <10^7^ cells or 15 mg wet weight tissue.^415^ Combined with semiautomated and high-throughput pipelines, large-scale immunopeptidomics comparing hundreds of clinical samples are now feasible.^416,417^ Modified immunopeptides of noncanonical origin can be also readily identified.^388,418^ As MS has become a standard analytical tool in life sciences laboratories, immunopeptidomics has gradually transitioned from experiments performed by a handful of experts to routine practices across the field. With the continuous evolution of MS techniques, especially in single-cell proteomics,^419^ we anticipate it may soon be possible to map the disease immunopeptidome at the cellular level.

#### 5.3.1 Data Analysis for Immunopeptidomics

Early practices in immunopeptidomics employed similar analysis pipelines as traditional proteomics. The quality of analysis is determined by how well the detected spectra match with ones generated *in silico*.^417,420^ However, applying this criterion to MHC peptides raises several challenges: (i) mutant or spliced peptides are often not included in standard database searches.^389^ (ii) Unpredictable fragmentation patterns occur for nondigested peptides. (iii) There is often low confidence in sequence identification. To address these challenges, several workarounds have been developed (Figure 8B). Targeted databases (e.g., SpectMHC^421^) can be constructed from existing data sets or through motif-driven predictions (e.g., MHCquant^422^) to improve search accuracy. However, these approaches may be biased toward well-studied alleles (e.g., HLA-A*02:01) due to data availability. Recently, packages such as the PEAKS studio have employed deep-learning-based spectral searching and de novo sequencing to enhance peptide identification, resulting in approximately a 2-fold improvement.^423,424^ Additionally, data-independent acquisition (DIA) mode has been utilized to enhance accuracy and peptide identifications.^391,425^ Using DIA, Bassani-Sternberg and co-workers identified thousands of high-confidence MHC peptides from as few as 10^6^ cells.^426^ This coevolution of analysis algorithms and sensitive mass spectrometers will further enhance our ability to profile the immunopeptidome.

In addition to peptide identification, algorithms are also needed to assign peptides to specific MHC alleles. Establishing the presenting allotype for detected peptides is challenging due to the polymorphism and isolations of MHC molecules.^391,427^ A popular solution is NetMHCpan,^428,429^ a neural network-based algorithm that leverages additional biochemical information (e.g., peptide length, motif preferences, existing databases) to assign alleles for input sequences. Researchers can also use NetMHCpan and its successors to forecast all possible segments from a protein that can be displayed as pMHC by a given allele. While convenient, these analyses are not perfect. Because the algorithms rely heavily on pre-existing data, predictions for understudied alleles are often poor.^420^ To tackle this issue, Wu and collages experimentally profiled 95 monoallelic cell lines and generated a reference database for training predictive algorithms.^430,431^ These data also revealed binding motifs for a large collection of MHC allotypes across the human population.^432^ Many submotifs, both unique and shared, exist across multiple alleles. Distinct motifs were also found within peptides of different lengths, suggesting that a sophisticated control mechanism underlies which peptides get presented. Finally, the refined data set can be integrated with DDA immunopeptidomics protocols to aid peptide identification, resulting in approximately a 2-fold improvement.^430^

#### 5.3.2 Quantification for Immunopeptidomics

Beyond peptide identification, quantification – estimating copies-per-cell – is critical for studying the immunopeptidome. Different relative and absolute quantification proteomic workflows have been implemented for this purpose, each with its own advantages and disadvantages due to the unique properties of MHC peptides (Figure 8C).^391,433^ Label-free quantification (LFQ) has been used to study how stimulation (e.g., IFN-γ, infection, etc.) alters the immunopeptidome and antigen presentation.^417,434^ Despite the ease of implementation, LFQ accuracy can vary due to poor agreement in peptide identification between samples (approximately 40% between biological replicates).^433^ Stable isotope labeling by amino acids in cell culture (SILAC) can improve run-to-run viability but faces challenges in choosing which labeled amino acids are best suited.^435,436^ Common residues from tryptic digests (e.g., lysine and arginine) are often absent in MHC peptides, leading to sparse labeling. Anchor residues (e.g., valine for HLA-A*02:01) can be used, but this is restricted to selected alleles. SILAC with tandem mass tags (TMT) has also been implemented for multiplex quantification.^437,438^

In addition to relative quantification, determining the absolute copies-per-cell for a given pMHC is possible with internal calibration using exogenous peptide standards.^404,439,440^ The timing to introduce peptide standards is crucial, though, and many protocols often fail to account for losses during pMHC extraction.^433^ To address this, White and co-workers developed hipMHC, where a synthetic, UV-exchangeable pMHC standard is used to mitigate peptide losses during processing and labeling.^441,442^ In addition to improving quantification accuracy, hipMHC can achieve absolute determination and provide copies-per-cell for a given pMHC when used with built-in calibration curve. This method is also compatible with multiplexing using TMT.^441^ Application of more sensitive detection techniques (e.g., selected or multiple reaction monitoring) has also enabled more accurate quantification of peptides in low-input settings.^391,443^ However, with the myriad of quantification strategies available, there is no clear consensus on the best practices for quantitative immunopeptidomes.^420^ Establishing such consensus will be critical for the community for future sharing and cross-comparison of data sets.

#### 5.3.3 Multiomics with Immunopeptidomics

Immunopeptidomics techniques have been integrated with other state-of-the-art proteogenomics to study the antigen presentation process.^409,420^ The introduction of Ribo-seq has led to a paradigm shift in immunopeptidome analysis. Ribo-seq is a technique that provides information on the polypeptides actively transcribed by ribosomes^444^ (a more detailed review can be found in reference (445)). When paired with immunopeptidomics, Ribo-seq can directly inform on the source of MHC peptides.^446,447^ These studies have also shed light on the mysterious inconsistencies between immunopeptidomes and transcriptomics. In a recent study from the Wu and Regev groups, it was demonstrated that the unexpected pool of MHC peptides is associated with novel or unannotated open reading frames (nuORFs).^448^ Additionally, nuORF peptides are immunologically active and potential targets of immunotherapy. It has also been postulated that nuORFs are more likely to contribute MHC ligands as they are more rapidly and efficiently degraded. This suggests that there remains a large portion of the immunopeptidome yet to be discovered via traditional MS methods.^449^ This is further reflected in a recent comprehensive study that profiles the immunopeptidome in combination with the acetylome, phosphoproteome, ubiquitylome, and proteome in tissue samples.^418^ Strikingly, only about 30% of sources contributing to an MHC peptide are found in the proteome, and only 26% of ubiquitylated proteins give rise to an MHC peptide. This mismatch further suggests that the immunopeptidome should be treated as its own special ″ome″ within the cell, rather than as a mere extension of the intracellular proteome.

### 5.4 Discovery of Disease-Associated Immunopeptidome

Immunopeptidomics not only reveals the basic principles of antigen presentation but also uncovers actionable targets for therapeutic development. The MHC peptide repertoire conveys information on cellular state in response to perturbations, both inside and outside the cell, particularly in cancer.^446,450^ Genomic mutations can give rise to mutant peptides, or neo-antigens, presented by MHC molecules on the cell surface.^451,452^ Oncogenic events can lead to dysregulation in translation and transcription, resulting in the display of noncanonical peptides.^446,453^ Many HLA molecules are also used as predictors for risk and protective alleles in various diseases.^454,455^ The clinical relevance of the immunopeptidome strongly motivates comprehensive interrogation under disease contexts. MS-based immunopeptidomics techniques have catalyzed the discovery of disease-associated alterations. However, the complexity of the peptide repertoire necessitates innovative integration with other disciplines such as bioinformatics, proteogenomics, and engineering. Below, we will discuss recent technologies in uncovering disease-associated immunopeptidomes.

#### 5.4.1 Prediction Algorithms for Antigen Discovery

**Figure 9:**
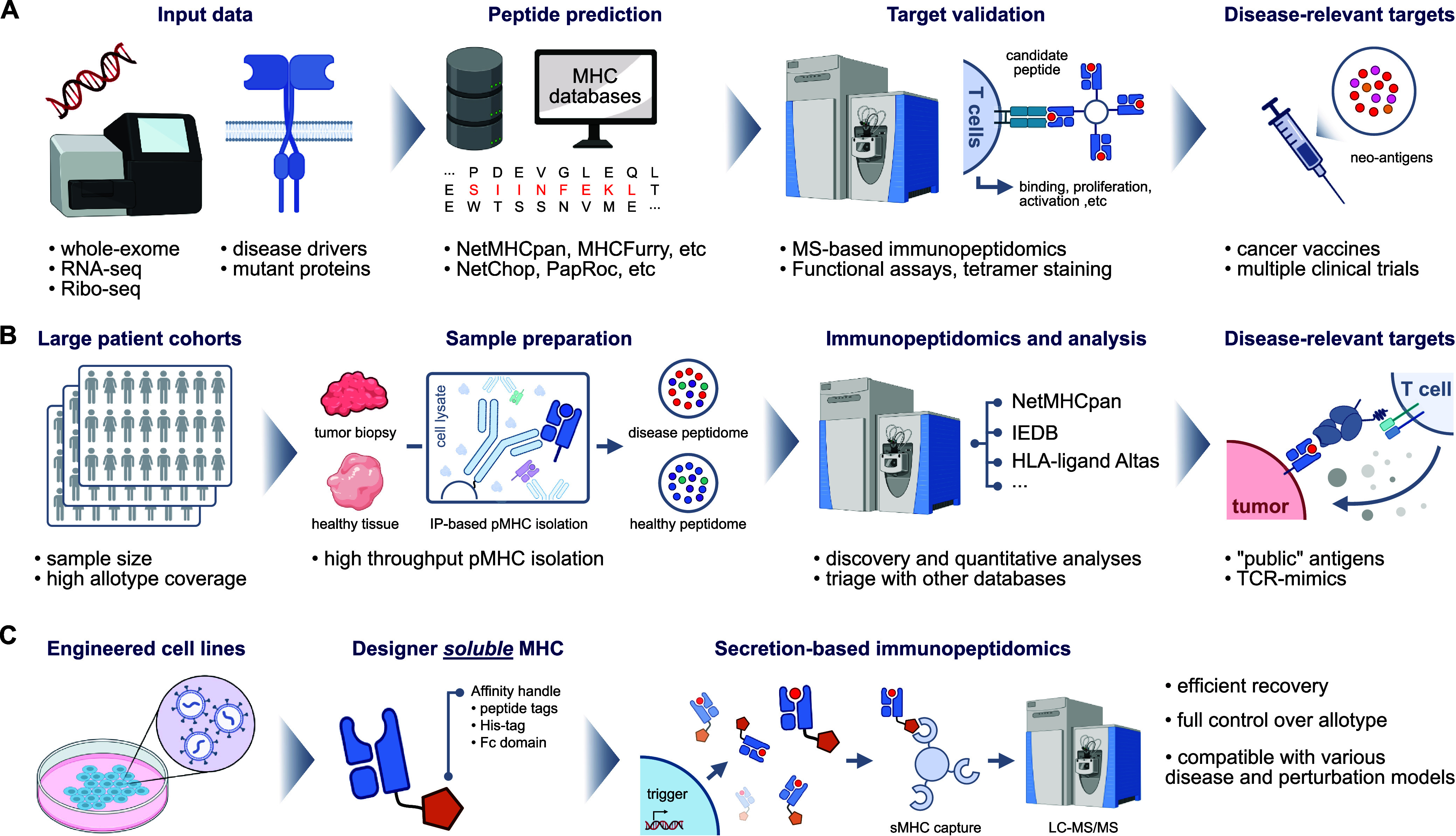
Platforms for mapping disease-induced immunopeptidome. (A) Diseased-associated pMHCs can be inferred from sequencing data. The predicted ligands required additional validation via functional assays and MS-based immunopeptidomics. This approach has yielded many MHC sequences for the development of cancer vaccines. (B) Actionable pMHCs can be identified from population-scale immunopeptidomics. These analyses seek sequences that are detected across large patient cohorts. Consequently, targets identified from these data sets are amenable for developing “off-the-shelf” agents. (C) Identifying allele-specific pMHCs is feasible with secretion-based platforms. Cells are engineered to secrete pMHCs of interest to enable detection of rare, disease-associated peptides that are less abundant. Portions of this figure were created with BioRender.com.

Early studies on the immunopeptidome hinted the possibility of mutant epitopes displayed by MHC molecules.^452,456^ This observation, coupled with the interest in personalized medicine,^457^ has spurred the development of numerous algorithms for predicting antigenic peptides from whole exome sequencing data (Figure 9A).^452,458^ Mutant sequences from cell lines and patient tumors are analyzed with software such as NetMHCpan to generate lead pMHCs for downstream validation using immune activity or mass spectrometry. Neo-antigens have been identified from tumors bearing difficult-to-drug drivers (e.g., TP53, KRAS, etc).^8,452,459,460^ Many vaccine formulations based on the predicted sequences are currently being evaluated in late-stage clinical trials.^461^ Despite some success, the “hit-rate” of prediction-based discovery remains low, and predicted peptides are rarely found on the surface of diseased cells.^462,463^ Further complicating the issue, many of the predicted peptides are not immunogenic.^373^ One potential explanation is the over-reliance on binding-based models, which overlook other crucial steps in antigen presentation, such as peptide processing and transport.^462^ Improvements have been introduced by considering complex stability (NetMHCstab^464^), susceptibility to proteasome cleavage (NetChop^465^), and enzyme processing (PAProC^466^). By integrating one or more filters with the binding-based models,^467^ better performance can be achieved in predicting bona fide immunogenic peptides.

#### 5.4.2 Genetic Approaches for Antigen Discovery

Experimental methods can be combined with antigen prediction to accelerate identification of immunogenic peptides. With advances in genetic screenings, large pools of potential peptide sequences (generated from prediction or sequencing data) can be introduced to cells. Any potential non-MHC ligands are eliminated as only folded complexes are displayed on the cell surface.^244,468^ These cells are then used in downstream functional assays and immunopeptidomics for antigen and T cell receptors (TCR) discovery. In one recent example, Elledge and co-workers reported an optimized cell platform (EpiScan) that enables large scale, targeted peptide discovery with user-defined allotype and biological context.^469^ Interestingly, this approach could identify MHC peptides enriched with cysteines – a severely underrepresented phenotype in existing immunopeptidome data sets. Such approach is very powerful as it enables TCR identification without prior knowledge of the immunogenic epitope. Rose and co-workers have shown that it is possible to survey potential epitopes from 47 cancer drivers simultaneously using a linked polyantigen cassette based on computation and empirical data.^470^ Using the same platform, they subsequently isolated TCRs capable of eliciting immune response against target pMHCs shared among the cancer mutations. This combination of neo-antigen prediction and functional screens will further bolster our ability to uncover therapeutically relevant pMHCs-TCR combinations.

#### 5.4.3 Large Scale Immunopeptidomics for Antigen Discovery

Performing immunopeptidomics across dozens of tumor specimens and cell lines have also contributed many disease-associated pMHC targets.^420^ Instead of focusing on neo-antigens unique to individuals, these studies seek shared antigens by comparing the immunopeptidome from large collections of healthy and disease sources (Figure 9B). Several disease types have been investigated, including breast and ovarian cancers, melanoma, and acute myeloid leukemia.^380,408,471,472^ In all cases, shared antigens were identified, but the hit rate was low. Interestingly, many peptides identified in tumor samples were also found in healthy tissue, suggesting the need for disease-specific controls or filters. In rare cases, shared, post-translationally modified (PTM) peptides can be observed.^473,474^ Certain PTMs, such as phosphorylation, have been suggested to enhance stability over unmodified peptides and are more likely to be found in the immunopeptidome under aberrant phosphorylation.^475^

Quantitative immunopeptidomics can further help prioritizing targets. Riley and co-workers recently identified a spliced peptide from collagen COL6A3 by analyzing ∼ 1,500 tumor and normal tissue samples.^476^ Interestingly, this peptide was observed across 55 tumors with diverse tissue origins and in healthy tissue, albeit at a much lower copies-per-cell. Conducting immunopeptidomics at scale, however, remains limited to experts specializing in immunopeptidome research. To address this issue, leaders in the field have launched the Human Immunopeptidome Project (HIPP) with the goal of comprehensively mapping the pMHC repertoire and developing robust technologies that are easily accessible to researchers.^420,477^ The HIPP also launched the systeMHC atlas project as an open-source repository for depositing data from different experimental settings.^425^ The growth of these community-driven efforts will accelerate large-scale comparisons of different cell models, disease types, and patient cohorts.

#### 5.4.4 Secretion of pMHC for Antigen Discovery on Specific MHC Alleles

Immunopeptidomics focusing on secreted MHC molecules can also reveal disease-associated markers and targets with allele-specific resolution. Cells can naturally shed or be engineered to secrete pMHCs into the extracellular milieu.^391^ Continuous enrichment of soluble complexes can enable sensitive detection of low abundance complexes (Figure 9C). The secreted method was originally adopted by Hildebrand and co-workers to establish binding motifs for HLA-B*15:01, as it was the only way to isolate MHC peptides on a large scale.^478^ In 2002, Admon and co-workers demonstrated the possibility of using secreted methods to analyze antigen presentation in breast, ovarian, and prostate cancer cell lines.^479^ A key feature of secreted immunopeptidomes is their close resemblance to the membrane-bound counterpart.^480^ Additionally, naturally shed sMHCs can be detected from patient serum and are present at higher concentration than tumor bound ones.^481^ Profiling the secreted repertoire can provide a noninvasive way to screen for patient compatibility for HLA-targeting therapeutics. Despite the potential, methods to engineer and profile secreted MHC remain limited. Many of the original secreted protocols were designed to work with impractically large amounts of input material.^478,479,482^

More recent work from the Strong laboratory and our group have introduced biochemical tags to sMHC to facilitate ease of secretion and purification of specific MHC alleles containing their immunopeptidomes.^483,484^ Strong and co-workers generated sMHC by replacing the transmembrane domain with a C-terminal poly histidine tag for isolation (SCD). The SCD was stabilized by linking the MHC heavy chain and β2m as a single fusion protein.^483,484^ In our case, we developed constructs featuring the ectodomain of only MHC heavy chain fused to the human Fc domain. Unlike the SCD, the MHC-Fc fusion heterodimerizes with endogenous β2m during biosynthesis. The presence of the Fc facilitates efficient expression and purification, and also reduces the input requirement by providing extra stability.^483,484^ We demonstrated the utility of this method by profiling the immunopeptidome of several cancer cell lines under different physiological states including senescence and hypoxia. One caveat of sMHC is the incompatibility with fresh tissue and primary cell lines. For this reason, we anticipate that the secreted platform will be used alongside with traditional immunopeptidomics to accelerate target discovery. For instance, the secreted platform could be used to profile potential targets under the confine of a specific disease context (e.g., oncoprotein activation, microbial/viral infection, dysregulated cell states, etc). This will provide a focus-list of pMHCs for further validation on clinically relevant specimens via traditional immunopeptidomics. The merger of these two approaches will thus will further bolster our ability to study and identify disease-relevant pMHCs.

#### 5.4.5 Technology for Capturing Dynamic Changes within Disease Immunopeptidome

So far, efforts to map the disease immunopeptidome have primarily focused on bulk tumor measurements, providing only a snapshot of cancer-specific antigen presentation. Early studies on the impact of interferon on the immunopeptidome revealed that the peptide repertoire is dynamic and responsive to cytokine stimulation.^417,485^ Later studies on ribosomal subunits and mTOR inhibition demonstrated that the immunopeptidome is regulated by a multitude of biochemical networks and metabolic events.^486^ These studies provide further evidence of the plasticity of the MHC peptide and the potential for dynamic changes during disease development. Studying these changes is challenging, as alterations can occur in both peptide identity and abundance levels.^391,433^ For instance, White and co-workers used hipMHC to reveal that MEK inhibition induced the display of novel sequences and upregulated a subset of existing ones, with some sequences detected over 1,000 copies per cell. Interestingly, many of these changes are not correlated with changes in transcripts or ubiquitylation levels.^442^ A recent study by Tyler and co-workers reported similar observations in lung adenocarcinoma immunopeptidomes.^487^ The authors of this study also developed a mouse model where antigen presentation can be dynamically controlled with inducible Cre recombinase expression. This model has the potential to enable immunopeptidome profiling across multiple stages of disease and reveal additional opportunities for therapeutic intervention.

### 5.5 Perspectives and Outlook

Since the discovery that MHC peptides are key ligands for eliciting T cell responses, our understanding of the immunopeptidome has evolved tremendously. A myriad of mechanisms can contribute novel peptides to the immunopeptidome beyond highly abundant intracellular proteins. With advances in mass spectrometry and bioinformatics, we can now map the peptidomes of cells and even tissues under various biological contexts. Concomitant improvements in isolation and sample preparation protocols will be needed in the future to push the boundary in depth and throughput for immunopeptidomics. The search for disease-associated peptides has also spurred the development of new tools featuring techniques from engineering, genetic screens, and other multiomics platforms. Such integrated platforms further reveal how the immunopeptidome can change in response to pathological transformation. Looking ahead, we anticipate the scope of immunopeptidomics will further expand with the introduction of single-cell proteomics, data-independent acquisition, and machine learning-based analysis pipelines.

Beyond profiling the immunopeptidome, strategizing the optimal way to therapeutically exploit disease-associated pMHCs is another major goal of the field. Many modalities targeting pMHCs have been developed, ranging for cancer vaccines, adoptive cell transfer, TCR- and antibody-based biologics, and more recently, CAR-T cells (excellent reviews on these topics can be found in references^427, 452, 488^. These drug discovery efforts are projected to grow exponentially in the next decade due to the recent FDA approval of Tebentafusp, a soluble TCR mimic that recruits T cells to attack tumors in a pMHC-dependent manner.^489^ Despite the excitement, the key determinants of efficacy and toxicity remain poorly characterized for pMHC-targeting modalities. Many of the rules for targeting cell surface proteins may not apply due to the unique characteristics of the immunopeptidome and MHC molecules. The extremely low expression levels of pMHCs present challenges for therapy: (i) low target expression confines the options for immuno-targeting using CAR-T or bispecific T-cell engagers (ii) the potency of these modalities requires knowing that the target is present on disease and not healthy tissue. (iii) the diversity of pMHC to contain closely related peptides stresses the need to exceedingly specific antibodies or TCR mimetics. Nonetheless, the pMHC discovery field has dramatically expanded the target space for new therapies as evidenced by the burgeoning number of start-up companies in this area. Overall, the rapidly improving technologies for allele-specific and targeted pMHC discovery coupled with new immunotherapies are making great strides in this exciting field.

## 6 Conclusions and Perspectives

The surfaceome presents vast opportunities for technological, biological, and therapeutic discovery. In the past decade, new engineered and chemical tools have allowed for enrichment methods to interrogate the surfaceome at much greater depth. Technologically, there have been great strides in mass spectrometry to increase accuracy and sensitivity. Additional informatics platforms are providing better annotation for surface proteins. This allows us to interrogate how the surfaceome changes from health and disease in terms of proteins expressed, their neighborhoods, their PTMs, and immunopeptidomes. These new targets, proteoforms, and complexes dramatically expand the therapeutic and biomarker opportunities for small molecules and biologics.

### 6.1 We Are Only Beginning to Scratch the Surface

Virtually all surfaceomic studies are conducted in 2D cell culture for the obvious advantage of material availability and reproducibility. One of the key new horizons will be to determine surfaceomes in disease and healthy tissues. Currently, the only validated technologies available for discovery in tissues are antibody- or RNA-based platforms. These have huge limitations. Antibodies generally bind to tertiary epitopes, requiring a folded protein target, which is destroyed upon paraffin-embedding for immunohistochemical (IHC) staining. It is rare to find IHC-compatible antibodies; they are expensive and lack comprehensive coverage. RNA-based hybridization for specific membrane proteins has the sensitivity and is cheaper but transcript levels are weakly correlative with protein levels. Importantly, most of the interesting surfaceome targets are generated post-translationally such as PTMs, protein complexes, and pMHCs. Thus, the onus is on the surfaceomics community to develop robust and sensitive methods for 3D and tissue-based surfaceomics. Tumors and healthy tissues are very heterogeneous and not renewable, which will challenge the field to analyze these samples using cell purification with small quantities as well as new informatics approaches to categorizing cell types. There have been some recent advancements in spatial proteomics on tumors to analyze specific regions of tumors and normal tissues from the Mann group^490^ and the Zhu and Qian groups.^491^ Deeper proteomics on whole tissue lysates can detect up to 1000 membrane proteins even among the much more abundant cytosolic proteins. Such studies obviate the need for surface enrichment but cannot localize the membrane protein to the cell surface or other internal membrane structures. Rapid progress is also being made in single-cell proteomics that could start to reach the sensitivity to detect surface proteins.

The new PLP approaches are in their early days of development but are already having an impact on discovering cell surface neighborhoods. Prior to this, the classic view of growth factor or small molecule cell signaling is that it is a vertical ‘bucket-brigade”. In this oversimplified vertical model, the ligand binds a single transmembrane receptor complex that ultimately causes a conformational change inside the cell that allows cytosolic proteins to engage, leading to functional changes inside the cell. These new surface PLP studies show there is a world of lateral signaling in the membrane that we are only beginning to appreciate.

Lastly, there is a tremendous interest in the structural biology and drug discovery community to define at atomic resolution the structure of the cell. This is a laudable goal and one of human genome-like importance. There are reasonable arguments that the surfaceome, the “edge-pieces” of the cell, with half the degrees of freedom should be a primary target for such endeavors as we are rapidly developing our ability to define surface interactomes and model their structures. Genome-wide sequencing led to the systematic identification of disease targets based on human genetics. The field of neo-protein epitopes is emerging where targets are identified, not at the gene or transcript level, but at the proteome level encompassing defined disease-associated PTMs, complexes, and dysregulated cellular and tissue location. Given the functional importance of the surfaceome for biology and therapy, we view surfaceomics as a critical piece of this quest for neo-epitope target discovery.
